# A New Multiparameter Model for Multiaxial Fatigue Life Prediction of Rubber Materials

**DOI:** 10.3390/polym12051194

**Published:** 2020-05-23

**Authors:** Rafael Tobajas, Daniel Elduque, Elena Ibarz, Carlos Javierre, Luis Gracia

**Affiliations:** 1 Department of Mechanical Engineering, University of Zaragoza, C/María de Luna, 3, 50018 Zaragoza, Spain; 2 i+aitiip, Department of Mechanical Engineering, University of Zaragoza, C/María de Luna, 3, 50018 Zaragoza, Spain; delduque@unizar.es (D.E.); carlos.javierre@unizar.es (C.J.); 3 i3A, Department of Mechanical Engineering, University of Zaragoza, C/María de Luna, 3, 50018 Zaragoza, Spain; eibarz@unizar.es (E.I.); lugravi@unizar.es (L.G.)

**Keywords:** life prediction, elastomers, rubber materials, multiaxial fatigue, damage parameter

## Abstract

Most of the mechanical components manufactured in rubber materials experience fluctuating loads, which cause material fatigue, significantly reducing their life. Different models have been used to approach this problem. However, most of them just provide life prediction only valid for each of the specific studied material and type of specimen used for the experimental testing. This work focuses on the development of a new generalized model of multiaxial fatigue for rubber materials, introducing a multiparameter variable to improve fatigue life prediction by considering simultaneously relevant information concerning stresses, strains, and strain energies. The model is verified through its correlation with several published fatigue tests for different rubber materials. The proposed model has been compared with more than 20 different parameters used in the specialized literature, calculating the value of the R^2^ coefficient by comparing the predicted values of every model, with the experimental ones. The obtained results show a significant improvement in the fatigue life prediction. The proposed model does not aim to be a universal and definitive approach for elastomer fatigue, but it provides a reliable general tool that can be used for processing data obtained from experimental tests carried out under different conditions.

## 1. Introduction

Rubber materials are widely used in the automotive and aeronautics industries due to their mechanical properties, such as wear resistance, deformation capacity, or vibration isolation [1,2,3,4]. Although they are present in components as important as tires [5,6,7], they are also used in the manufacture of other critical components, such as seals, insulating joints, and engine ducts [8,9,10]. All these types of components are generally subjected to fluctuating loads that, in many cases, compromise their durability due to the phenomenon of structural degradation known as material fatigue [11]. Fatigue is a major factor in mechanical design [12] as it still is one of the most uncertain and highly unpredictable failure mechanisms [13,14].

However, a large number of parameters influence the durability of rubber materials [15,16,17,18,19,20,21,22]. Thus, in order to design and manufacture reliable parts, the effective prediction of fatigue life still needs to be better understood and modelled.

In general, it is established that the fatigue failure of any component is divided into three stages: crack nucleation stage, crack propagation stage, and component failure. Although there are works in the literature that study the fatigue phenomenon of these materials during the crack propagation stage [23], this paper establishes its objective in the crack nucleation stage. This is because, in general, the fatigue life of the crack nucleation stage represents, at least, 90% of the total fatigue life of a component [24].

Currently, the prediction of the fatigue life of rubber materials faces two major difficulties. On the one hand, it is necessary to obtain a precise value of the stresses, strains and strain energies of the entire component to be analyzed, throughout the complete load history [25,26]. In order to do so, it is necessary to carry out the appropriate simulations using a sufficiently precise model that takes into account all the inelastic effects of the material: hyperelasticity, viscoelasticity, Mullins effect, etc. [27,28]. On the other hand, it is necessary to go through the corresponding fatigue tests and establish a fatigue parameter capable of correlating with the lifetime of each of the samples tested [29,30,31].

Fatigue damage parameters based on stresses have been widely used in the fatigue analysis for metallic materials with remarkable success [32]. They have also been applied to rubber materials [33,34,35], but without considering the essential differences in terms of mechanical behavior. In order to deepen the understanding of the fatigue behavior of rubber materials, Mars and Fatemi [36] conducted a literature study on the different fatigue damage parameters that had been studied for a fatigue analysis of rubber materials. They identified the maximum principal strain and the strain energy density (SED) [37] as the two most used parameters, and as the most capable ones for quantifying the damage in the nucleation phase. They also observed that other parameters related to strains had been used.

While the parameters related to strains have given acceptable results, it has always been believed that leaving aside the field of stresses can worsen the results because essential information is missing in the corresponding models. For this reason, the use of SED as a fatigue damage variable is the easiest method to take into account both strain and stress, and it has been widely used as well [38]. For rubber materials, such SED is estimated from a hyperelastic model fully defined in terms of strain.

As a conclusion from a complete literature review it could be established that, although there are many studies on the fatigue life of rubber materials, all of them provide prediction models valid only for the specific studied material and for the type of specimen used (i.e., strains and stress distributions according to geometry and applied loads). This way, it is not difficult to understand that an experimental test that favors fluctuation in a certain variable (i.e., maximum principal stress), makes that the variable that best correlates the fatigue life is that same variable (maximum principal stress). In this sense, it can be asserted that there is not an objective and independent parameter able to predict the fatigue behavior of elastomeric materials for multiaxial stress states.

In this work, the development of a new model in order to achieve the generalization of the nucleation fatigue problem [39,40] for this type of materials and for all types of load conditions is performed. This new model provides the state of science and industrial knowledge with a single model capable of predicting the durability of a component regardless of the rubber material used and the load state to which it is subjected.

A comparison is made between the fatigue parameters most used in the bibliography, and the proposed model, for two different rubber materials widely used in industry, more specifically in the automotive and aeronautics field, NBR (acrylonitrile-butadiene rubber) and the SBR (styrene-butadiene rubber) [41,42,43,44], and two different types of experimental tests. The 83 tests performed by Mars and Fatemi [45] with the NBR material and the 128 tests performed by Ayoub and collaborators [46] with the SBR material were used. Based on the results of these references, a unified fatigue model will be proposed (for both materials) that will improve the predictions obtained with the previously available parameters.

## 2. State-of-the-Art Review of Fatigue Damage Parameters for Rubber Materials

### 2.1. Fatigue Damage Parameters Based on Stresses, Strains, and Energy

Concerning stresses, Lu [47] used the maximum principal stress in diabolo type specimens. Abraham et al. [48] concluded that the value of the maximum principal stress could not be used in materials that do not crystallize. André et al. [49,50] also used amplitudes and mean values of normal stress [51]. Wang et al. [52] used the finite element method to calculate stress-based fatigue damage parameters, including the logarithmic principal stress and the Cauchy principal stress.

With respect to strains, Woo et al. [53] used the Green –Lagrange deformation. Suryatal et al. [54] selected the maximum principal strain. Currently, the maximum principal strain remains one of the most widely used fatigue damage parameters [55]. This parameter has a good correlation with the fatigue life for uniaxial tests and has been used for tensile and torsional tests as well, giving acceptable results. However, it does not predict the difference between simple and equibiaxial traction [56].

Finally, for energy parameters, Greensmith [57] started using the SED criterion as the parameter defining the onset of fatigue cracks. Roberts and Benzies [56] also used this parameter along with the maximum principal strain in uniaxial and equibiaxial fatigue tests. His work was discussed by Mars [58], obtaining as conclusion that both the SED and the maximum principal strain were two criteria that do not take into account the closing of the cracks, so they were not able to distinguish the effect that traction and compression had on these cracks.

### 2.2. Fatigue Damage Parameters Based on the Critical Plane

Cracking energy density (CED) has recently been proposed by Mars and Fatemi [59], constituting a novel and promising [60] fatigue critical parameter. It basically consists of the portion of deformation energy density used to generate a crack in a specific plane. Verron [61,62] also formulated a parameter based on a critical plane, assuming that intrinsic defects populate the elastomer material. His parameter was based on the rate of energy release in all possible planes of crack appearance.

Other energy-based critical plane fatigue parameters have been developed in the literature [63,64] and have had high relevance in metallic materials. Although they have not been used in elastomers, probably due to their low correlation with experimental results, the good outcomes offered in other materials make them worthy of being used and taken into account in this paper: Fatemi-Socie [65]; Smith, Watson, and Topper [66]; Liu [67]; Findley [68]; Brown-Miller [69]; and Wang-Brown [70].

#### 2.2.1. Cracking Energy Density (CED)

Mars and Fatemi [59] define the CED in terms of its increment *dW*_c_ in the plane of failure, as shown in Equation (1):

(1) $$dW_{c}=r^{T}\cdot \sigma \cdot d\varepsilon \cdot r$$

where *r* is a unit vector that defines the normal vector to the plane of interest, *σ* is the stress tensor, and d**ε** is the strain increment tensor.

It should be noted that, in the case, of uniaxial tensile stress, there is only one failure plane and the CED coincides with the SED.

#### 2.2.2. Fatemi-Socie Parameter

Fatemi-Socie [65] proposed a model of shear damage under the premise that the dominant fracture plane, or critical plane, is the orientation plane θ that maximizes the parameter formulated in Equation (2):

(2) $${\left[\frac{\Delta \gamma }{2}\left(1+k_{FS}\frac{\sigma_{n,max}}{\sigma_{y}}\right)\right]}_{\max\theta }\;$$

where Δ*γ*/2 is the maximum amplitude of shear strain in a plane θ, *σ*_n,max_ is the maximum normal stress in that plane, *σ*_y_ is the elastic limit of the material, and *k*_FS_ is a constant of the material.

#### 2.2.3. Smith–Watson–Topper Parameter

Smith, Watson, and Topper [66] proposed that the critical fatigue plane corresponds to the orientation plane θ, which has the maximum normal stress (the maximum principal stress). According to the authors, the parameter that defines the fatigue life of the material is the product of the maximum normal stress and the maximum principal strain range, as shown in Equation (3):

(3) $$max_{\theta }\left(\sigma_{n}\right)\frac{\Delta \varepsilon_{1}}{2}\;$$

where *σ*_n_ is the normal stress in a plane θ, and *ε*_1_ is the maximum principal strain range.

#### 2.2.4. Liu I and Liu II Parameters

In 1993 Liu [67] formulated a model in which two possible failure planes were considered: one failure due to normal stresses and strains, and another one due to shear stresses and strains. In the first one, the critical plane is defined by that plane where the product of normal stress and strain is maximum, and the fatigue life is determined by that product plus the product of shear stress and strain in that plane. However, the second possible plane of failure is defined by that plane where the product of shear stress and strain is maximum, and the fatigue life is determined by that product plus the product of normal stress and strain in that plane. They are defined in Equations (4) and (5).

(4) $$\Delta W_{I}=\left(\Delta \sigma_{n}\Delta \varepsilon_{n}\right)max_{\theta }+\left(\Delta \tau \Delta \gamma \right)\;$$

(5) $$\;\Delta W_{II}=\left(\Delta \sigma_{n}\Delta \varepsilon_{n}\right)+\left(\Delta \tau \Delta \gamma \right)max_{\theta }\;$$

#### 2.2.5. Findley Parameter

From observations on the orientation of fatigue cracks in steel and aluminum, Findley [68] studied the influence of normal stress acting on the plane of maximum shear stress. On this concept, he formulated a critical plane model, which proposes as fatigue crack plane the orientation plane θ with the maximum damage parameter formulated according to Equation (6):

(6) $$max_{\theta }\left(\tau_{a}+k_{F}\sigma_{n,max}\right)\;$$

where *τ*_a_ is the amplitude of the shear stress in a plane θ, *σ_n,max_* is the maximum normal stress in that plane, and *k*_F_ is a constant parameter property of the material.

#### 2.2.6. Brown –Miller Parameter

As with Findley’s parameter for high cycle fatigue (HCF), Brown and Miller [69] proposed that, in the characterization of the fatigue phenomenon, both the shear and the normal stress in the plane of maximum shear stress should be considered. According to the authors, cyclic shear stresses help nuclear cracks, and normal stress helps their growth. Later, Kandil, Brown and Miller [71] proposed a simplified formulation of this theory as defined in Equation (7):

(7) $$\Delta \hat{\gamma }={\left(\Delta \gamma_{max}^{\alpha }+S\Delta \varepsilon_{n}^{\alpha }\right)}^{\frac{1}{\alpha }}\;$$

where *γ* is the equivalent angular distortion range, and *S* is a material constant that represents the influence of normal strain on the growth of cracks in the material. More recently, Wang and Brown [70] added a medium stress term to the formulation and assuming *α* = 1, so the equivalent shear stress amplitude was formulated as shown in Equation (8):

(8) $$\frac{\Delta \hat{\gamma }}{2}=\frac{\Delta \gamma_{max}}{2}+S\Delta \varepsilon_{n}^{\alpha }\;$$

where *γ*_max_ is the maximum angular distortion range, and *ε*_n_ is the normal strain range in the plane experienced by the angular distortion range *γ*_max._

#### 2.2.7. Wang –Brown Parameter

Based on Brown –Miller’s criteria, Wang –Brown [70] defined the critical plane as the plane θ where the maximum of the Equation (9) is reached:

(9) $$max_{\theta }\left(\frac{\Delta \gamma }{2}+S\Delta \varepsilon_{n}\right)\;$$

where Δ*γ* is the angular strain range in the plane θ, Δ*ε_n_* is the normal strain range in the same plane θ, and *S* is a property of the material.

#### 2.2.8. McDiarmid Parameter

McDiarmid criterion is based on tensions, and according to the authors [72], the critical plane is the plane where the shear stress is maximum. The equivalent stress of McDiarmid is defined as shown in Equation (10):

(10) $$\sigma_{eq}=\frac{\Delta \tau_{max}}{2}+\frac{\tau_{f}}{2\sigma_{u}}\sigma_{n,max}\;$$

where Δ*τ_max_* is the maximum shear stress range, *σ_n,max_* is the maximum normal stress in the direction perpendicular to the Δ*τ*_max_ plane, *τ*_f_ is the limit of torsional fatigue, and *σ_u_* is the ultimate tensile strength of the material.

In view of the state of the art, and the complexity of finding a suitable model to characterize the fatigue behavior of different elastomeric materials, the aim of this work is the definition of a general multiparameter model called Fatigue Damage Multi-Parameter (FDMP), which is capable of correlating the fatigue life for elastomer materials, without underestimating the information provided by the different fatigue variables, include those magnitudes used by different authors in order to evaluate fatigue life for elastomeric materials (i.e., stresses, strain, energy, critical planes), and they have proved their reliability in conjunction with different types of experimental tests. The FDMP model does not aim to be a universal and definitive approach for elastomers fatigue, but provide a reliable general tool that can be used for processing data obtained from experimental tests carried out under different conditions.

## 3. Proposed Fatigue Damage Multi-Parameter (FDMP) for Multiaxial Fatigue Analysis

### 3.1. Proposed Fatigue Multi-Parameter

As the literature review has evidenced, the variables that affect the fatigue life of two different elastomer materials are also different, so there is no general parameter for these type of materials, and there is a research gap that the model proposed in this paper is intended to fill.

This work presents a general multiparameter model called the fatigue damage multi-parameter (FDMP). It is capable of correlating the fatigue life for any type of material, and more specifically for elastomer materials. The idea of this parameter is not to underestimate the information provided by other fatigue variables, even though these are not the ones that characterize this behavior. Traditionally, when it is established that a variable (for example, maximum main strain) defines the fatigue behavior of a material, we disregard the information provided by other variables (stress variables, energy variables or even other strain variables).

The parameter proposed in this paper (FDMP) aims to take into account “*n*” fatigue variables, with their amplitude and mean values, assigning each of them a pondered weight so that the sum of these values multiplied by their weights maximizes the correlation coefficient *R*^2^ with the real experimental data. The calculated weights maximize the value of R^2^ for a line defined in Equation (11):

(11) FDMP=A·Ln(Lifetime)+B 

The weights of each variable should be calculated from the maximum possible number of fatigue tests. Once the evolution of the “*n*” variables is known throughout a load history for all the tests, by means of an optimization algorithm, the weights of each one of the variables are obtained. The advantage of the proposed model lies not only in taking into account “*n*” variables, since the computational cost of the optimization algorithm can be high, but also in the fact that this parameter can be limited to at least one variable, thus having a classic model of fatigue.

The FDMP value is defined as shown in Equation (12):

(12) $$FDMP=\alpha_{1}\cdot N_{cycles-var1}\cdot var_{eq,\;1}+\beta_{1}\cdot var_{max,\;1}+\alpha_{2}\cdot N_{cycles-var2}\cdot var_{eq,\;2}+\beta_{2}\;\cdot var_{max,\;2}+\cdots +\alpha_{n}\cdot N_{cycles-var,\;n}\cdot var_{eq,\;n}+\beta_{n}\cdot var_{max,\;n}$$

where:

- α_i_: Weights for each variable (amplitude and mean value) taken into account
- *β*_i_: Weights for each variable (maximum value) taken into account
- *N*_cycles-var,i_: Number of cycles experienced by variable *i* during one load cycle.
- The value of var_eq,i_ is defined in Equations (13) and (14):
  (13) $$var_{eq,i}=var_{amp,\;i}\cdot {\left(1-R_{var,\;i}\right)}^{\gamma_{i}-1}\;$$
  (14) $$\;R_{var,\;i}=\frac{var_{min,\;i}}{var_{max,i}}\;$$
- *γ*_i_: Coefficient in order to take into account the mean value of each variable

For uniaxial fatigue, the load cycle and the cycle of each variable will be the same, and the value of *N*_cycles_ will be equal to 1. For the cases in which, *N* = 1, *β*_1_ = 0, and *γ*_1_ = 1, they will be like a classic fatigue model with only one variable and only taking into account its amplitude.

The critical fatigue variables that can be used with this model are unlimited. It must be taken into account that, with more critical fatigue variables, the optimization problem will be more significant. However, one of the advantages of this model is that any other fatigue model formulated in the future can be implemented within this model and, in combination with others can, as a minimum, improve the results obtained.

### 3.2. Methodology for Validation of the Proposed Model (FDMP)

In order to validate the proposed model, a comparison work was done with the classical models and the models explained in Section 2 for the Mars and Fatemi NBR experiments [45], and for the Ayoub SBR experiments [46] They are described in Section 5. Using the coefficient of determination *R*^2^, it was established which of all the models correlated best with the fatigue life of each of the experiments.

The methodology used for the comparison and validation between the models consisted of:

1. Numerical simulation of each of the tests for each of the specimens and each of the materials.
2. Obtaining and calculation of the evolution of 20 mechanical variables throughout a load cycle.
3. Calculation of the number of cycles, amplitude, maximum value, and average value of each variable for each load cycle.
4. Correlation of the calculated values with the fatigue life of each material and obtaining the parameter *R*^2^.
5. Calculation of weights and coefficients for the model proposed in this work for each of the test batteries.
6. Application of the proposed model, correlation with the fatigue life of each material and obtaining the parameter *R*^2^.
7. Comparison of the R^2^ parameters of all the fatigue variables considered.
8. The obtained results are analysed in detail in Section 7: Discussion.

### 3.3. Experimental Data for Model Validation

#### 3.3.1. Mars and Fatemi Experimental Data Description (NBR Tests)

**Figure 1:**
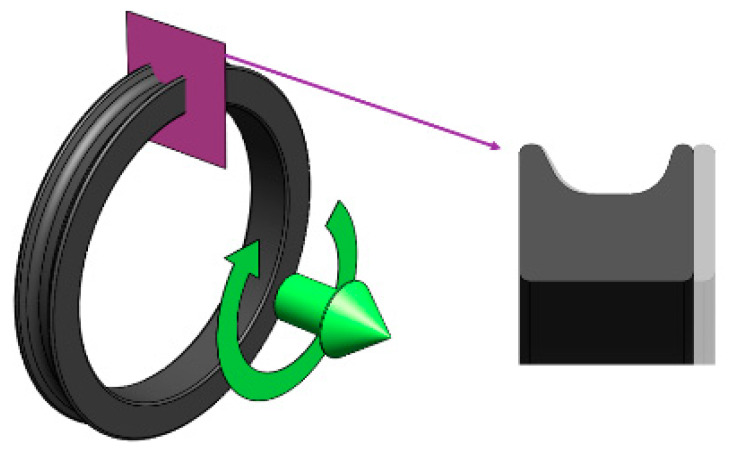
3D model for the calculation of stresses, strains and energies, based on Mars and Fatemi tests [45].

Mars and Fatemi [45] subjected a ring-shaped specimen to longitudinal deformations and torsions, in-phase and offset, at different amplitudes and mean values (Figure 1). A summary of the obtained results can be seen in Table 1. The full table is available as supplementary material, in Table S1.

#### 3.3.2. Ayoub Experimental Data Description (SBR Tests)

**Figure 2:**
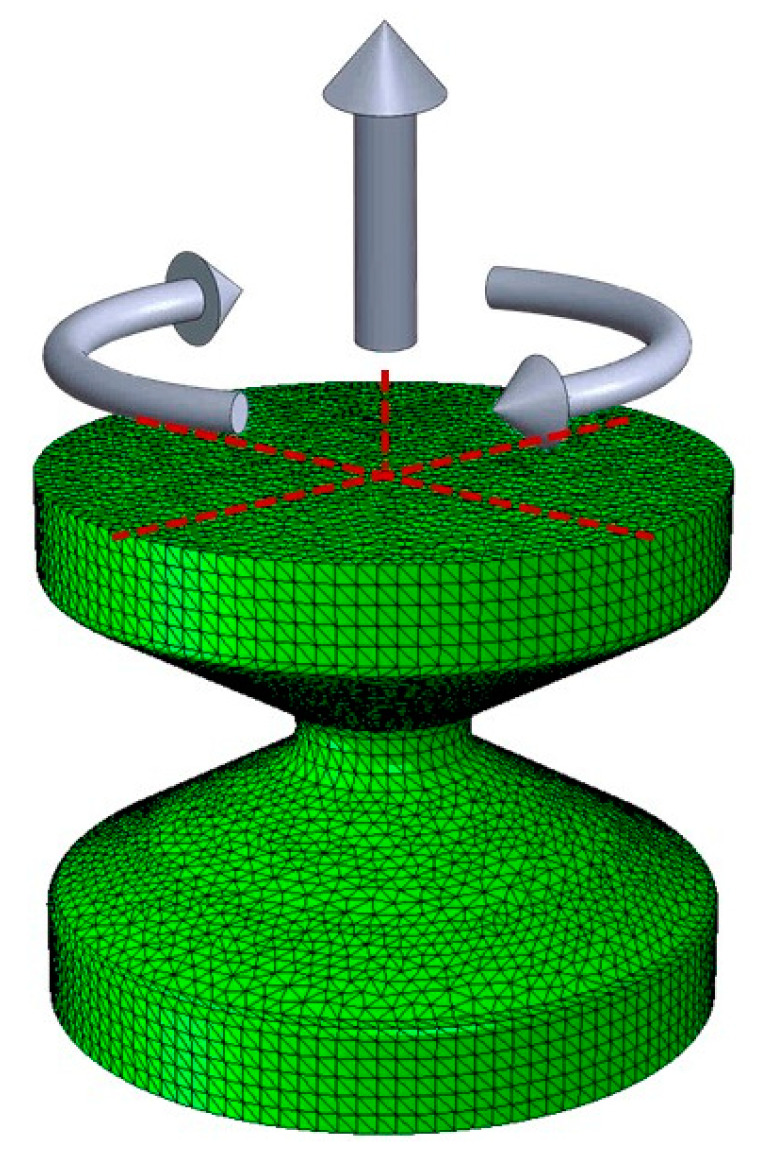
3D model for the calculation of stresses, strains and energies, based on Ayoub tests [46].

Ayoub [46] subjected a diabolo shape specimen to longitudinal deformations and torsions, in-phase and offset, at different amplitudes and mean values (Figure 2). A summary of the obtained results can be seen in Table 2. The full table is available as supplementary material, in Table S2.

## 4. Numerical Simulations and Results

### 4.1. NBR Numerical Simulations and Results

**Figure 3:**
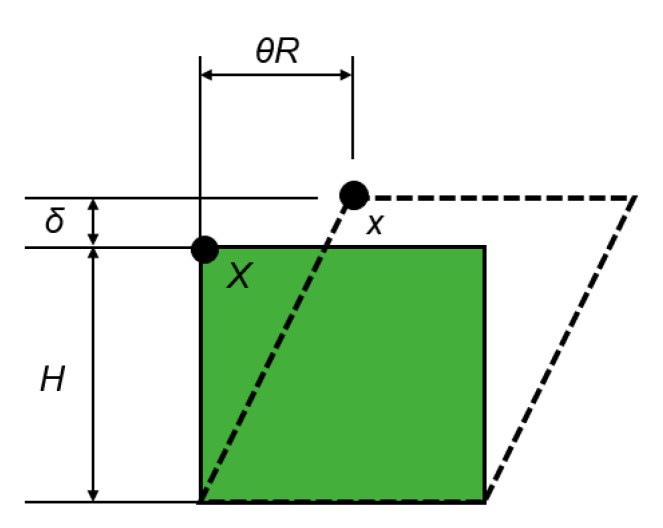
Original and deformed configuration of a material element subjected to axial and shear strains resulting from axial and torsional displacements *δ* and *θ*. Adapted from [45].

**Figure 4:**
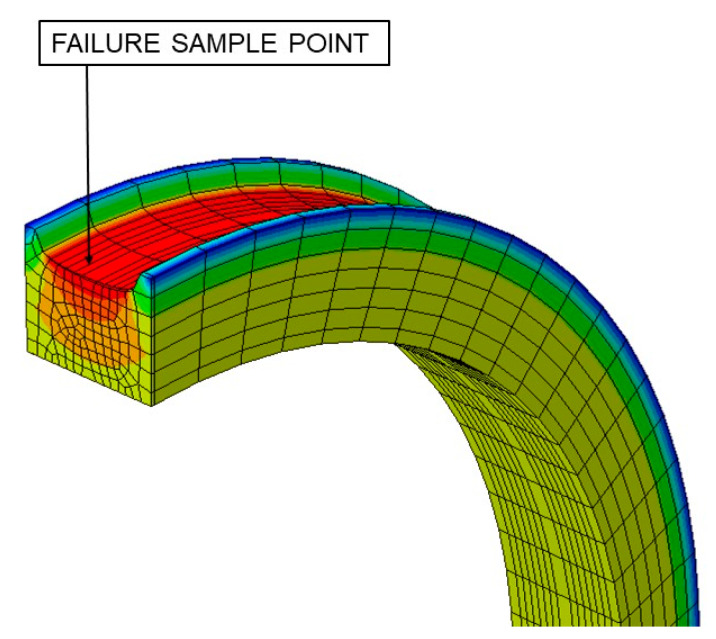
FEM simulation for test A1.

Mars and Fatemi [45] obtained the fatigue parameters values (stress, strain, and energy fields) by extracting the test conditions to a 2D model (Figure 3). In order to obtain more accurate values, the effects of multiaxial stresses have been taken into account in this work by the FEM (finite element method) simulations (Figure 4).

According to the authors, the material used in their tests belongs to the family of the elastomers whose behaviour is adjusted as a hyperelastic material that can be simulated by formulating the NeoHookean model [31], and as shown in Equation (15) with a constant value of *C*_1_ = 1.5 and *J* = 1.

(15) $$W=C_{1}\left(I_{1}-3\right)+\frac{1}{D}{\left(J-1\right)}^{2}\;$$

where *C*_1_ and *D* are material constants and *J* is the determinant of the gradient strain tensor F.

**Figure 5:**
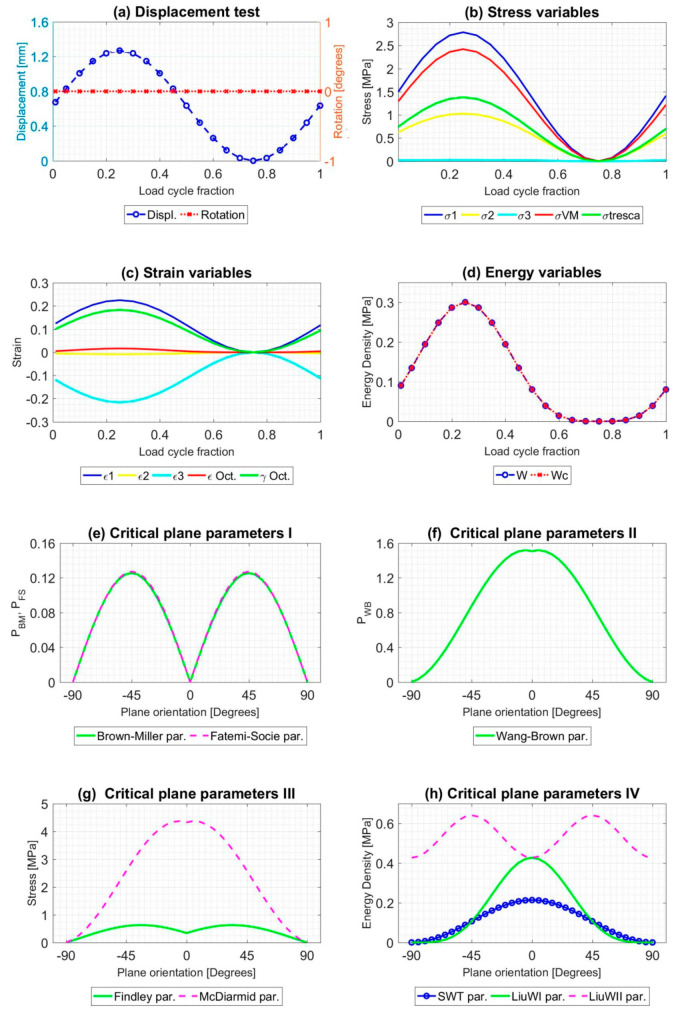
Result of the mechanical simulation of test A1 at the failure point of the sample caused by fatigue. Initial data from [45].

**Figure 6:**
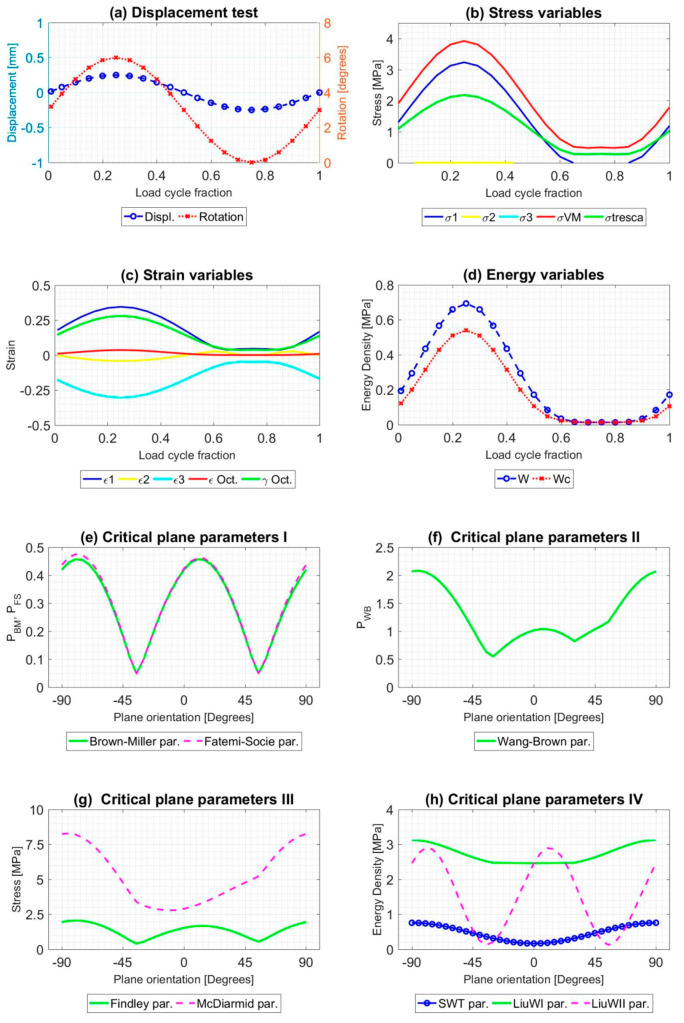
Result of the mechanical simulation of test B1 at the failure point of the sample caused by fatigue. Initial data from [45].

All the tests described were simulated during a load cycle, and mechanical variables were extracted. Results for A1 and B1 tests, described in Table 1, are shown in Figure 5 and Figure 6, respectively. Both figures provide plots in order to check the evolution of several variables: (a) Displacement [mm] and torsional rotation [degrees] imposed on the sample in a load cycle. (b) Stress variables [MPa]: principal stresses (*σ*_1_, *σ*_2_ and *σ*_3_), Von Mises stress (*σ*_VM_), Tresca stress (*τ*_max_). (c) Strain variables: principal strains (*ε*_1_, *ε*_2_ and *ε*_3_), octahedral strain (*ε*_oct_), octahedral angular strain (*γ*_oct_). (d) Energy variables [MPa]: Strain energy density (*W*), crack energy density (*W*_c_). In addition, the evolution of the critical plane parameters according to the angle of this plane concerning the maximum main direction are plotted: (e) Dimensionless parameters: Brown–Miller (*P*_BM_) and Fatemi–Socie (*P*_FS_). (f) Dimensionless parameter: Wang–Brown (*P*_WB_). (g) Parameters with stress dimensions: Findley and McDiarmid [MPa]. (h) Parameters with energy density dimensions: Smith–Watson–Topper (SWT), and Liu WI and Liu WII [MPa].

### 4.2. SBR Numerical Simulations and Results

Ayoub [46] characterized the material used in their tests by a hyperelastic model of Ogden (*N* = 2) which defines the stress-strain ratio by the variable SED (*W*) defined as:

(16) $$W=\overset{n}{\underset{i=1}{\sum }}\frac{2\mu_{i}}{\alpha_{i}^{2}}\left[\lambda_{1}^{\alpha_{i}}+\lambda_{2}^{\alpha_{i}}+\lambda_{3}^{\alpha_{i}}\right]$$

where *μ*_1_ = 5.25 MPa, *μ*_2_ = 1.52 × 10^−2^ MPa, *α*_1_ = 2.14 × 10^−1^, *α*_2_ = 4.06 and λ_1_, λ_2_ and λ_3_ are the principal stretches.

**Figure 7:**
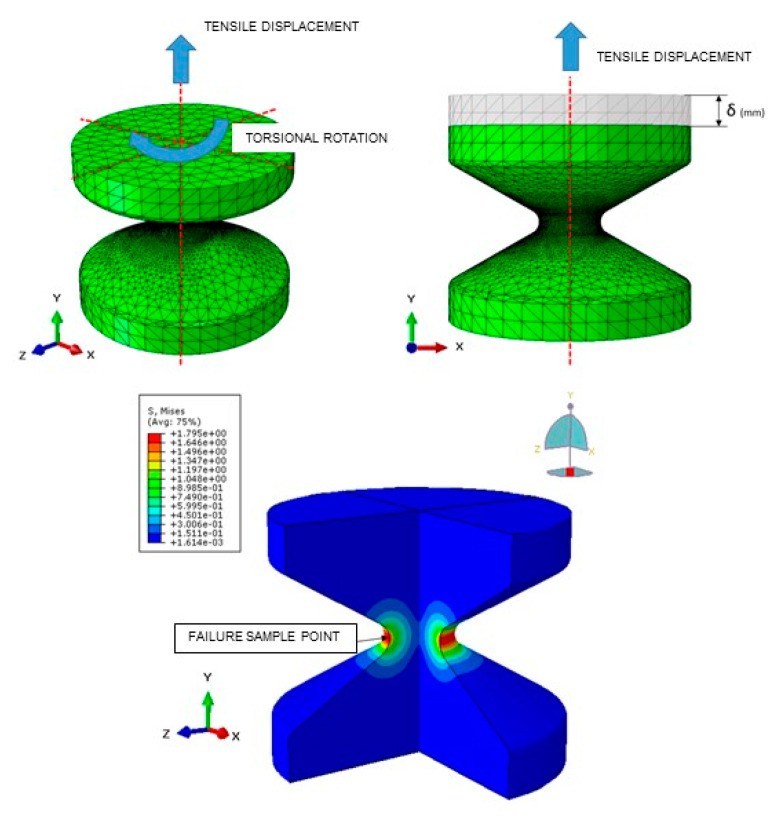
FEM model for test simulations.

**Figure 8:**
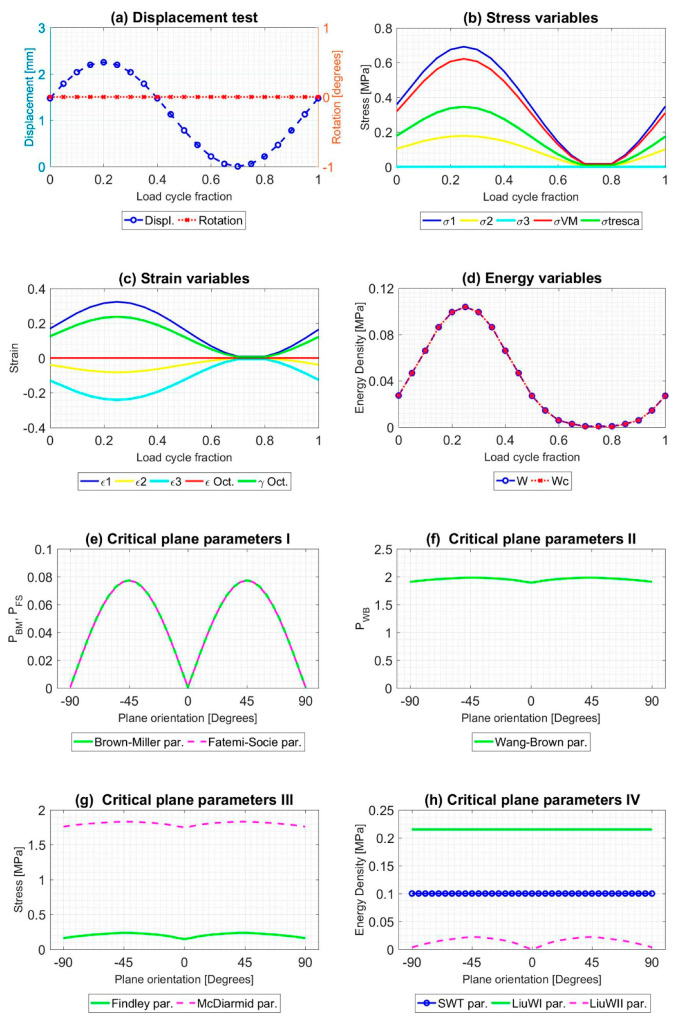
Result of the mechanical simulation of test A1 at the failure point of the sample caused by fatigue. Initial data from [46].

**Figure 9:**
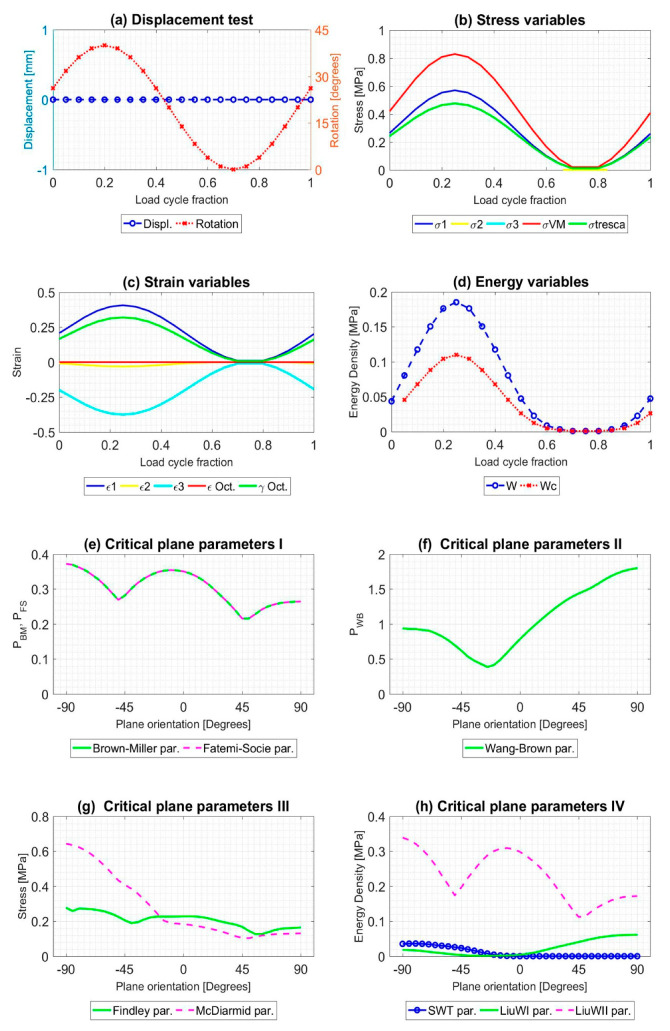
Result of the mechanical simulation of test B9 at the failure point of the sample caused by fatigue. Initial data from [46].

Based on these data, as shown in Figure 7, finite element simulations of each test were performed to obtain the history of classical fatigue parameters and all fatigue parameters defined in Section 2 for each load cycle:

All tests described were simulated during a load cycle, and mechanical variables were extracted in order to obtain the evolution of that. Results for A1 and B9 tests, described in Table 2, are shown in Figure 8 and Figure 9. As Figure 5 and Figure 6, Figure 8 and Figure 9 provide plots in order to check the evolution of several variables: (a) Displacement [mm] and torsional rotation [degrees] imposed on the sample in a load cycle. (b) Stress variables [MPa]: principal stresses (*σ*_1_, *σ*_2_ and *σ*_3_), Von Mises stress (*σ*_VM_), Tresca stress (*τ*_max_). (c) Strain variables: principal strains (*ε*_1_, *ε*_2_ and *ε*_3_), octahedral strain (*ε*_oct_), octahedral angular strain (*γ*_oct_). (d) Strain energy density (*W*) [MPa], Crack energy density (*W*_c_) [MPa]. In addition, the evolution of the critical plane parameters according to the angle of this plane with respect to the maximum main direction are plotted: (e) Dimensionless parameters: Brown-Miller (P_BM_) and Fatemi-Socie (P_FS_). (f) Dimensionless parameter: Wang-Brown (P_WB_). (g) Parameters with stress dimensions: Findley and McDiarmid [Mpa]. (h) Parameters with Energy Density dimensions: Smith-Watson-Topper (SWT), Liu WI and Liu WII [ MPa].

## 5. Fatigue Lifetime Correlation

Once the evolution of each of the mechanical variables at the failure points was obtained, the correlation of each of the variables and the fatigue lifetime of tests was established.

### 5.1. NBR–Fatigue Parameters Correlation

**Figure 10:**
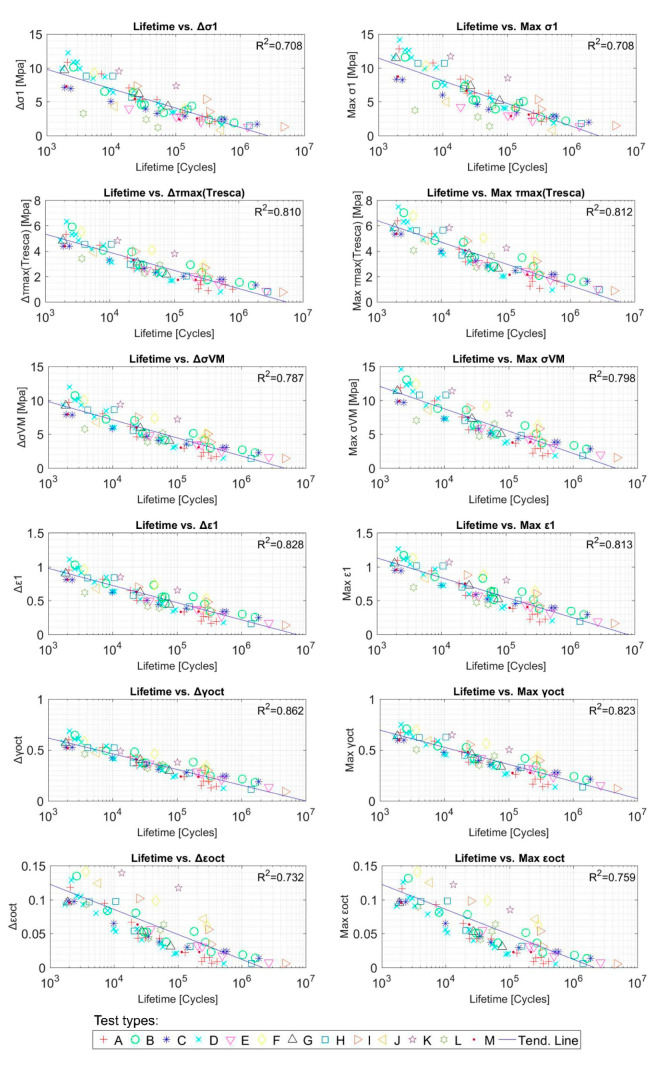

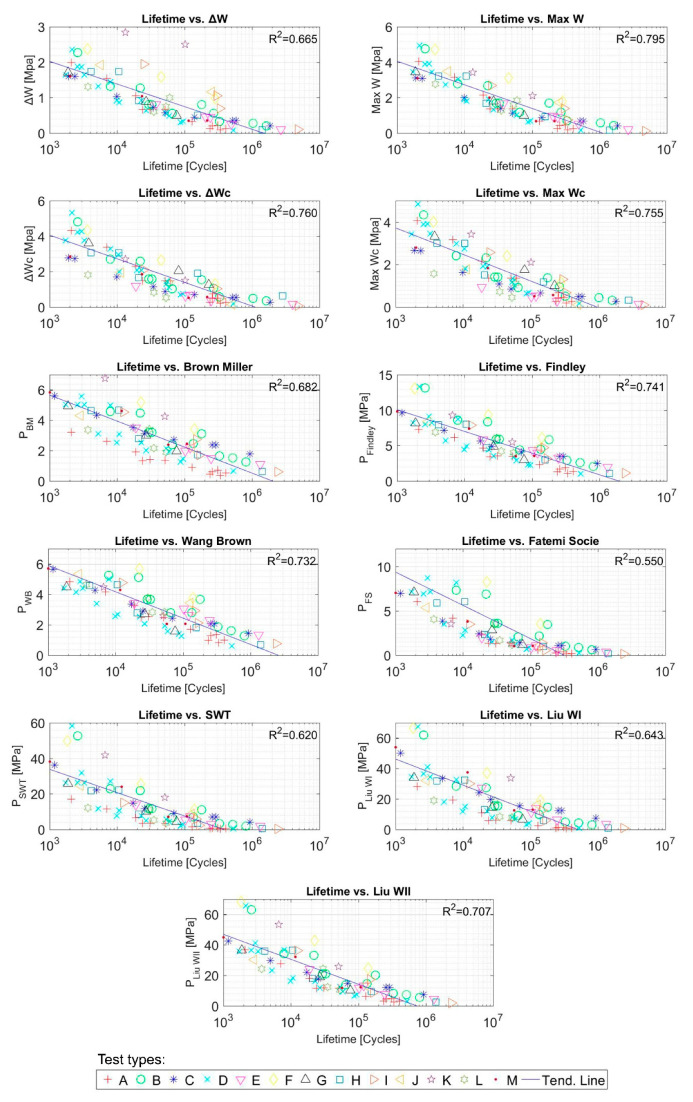
Fatigue parameters and tests lifetime [cycles] correlation for NBR tests. Initial data from [45].

Figure 10 represents the correlation between all of the studied mechanical variables versus the lifetime of the samples. In order to differentiate each type of test (see column 2 of Table 1) related by the authors with letters (A, B, …, M), these ones have been represented in plots by colours as referred in the legend.

### 5.2. SBR–Fatigue Parameters Correlation

**Figure 11:**
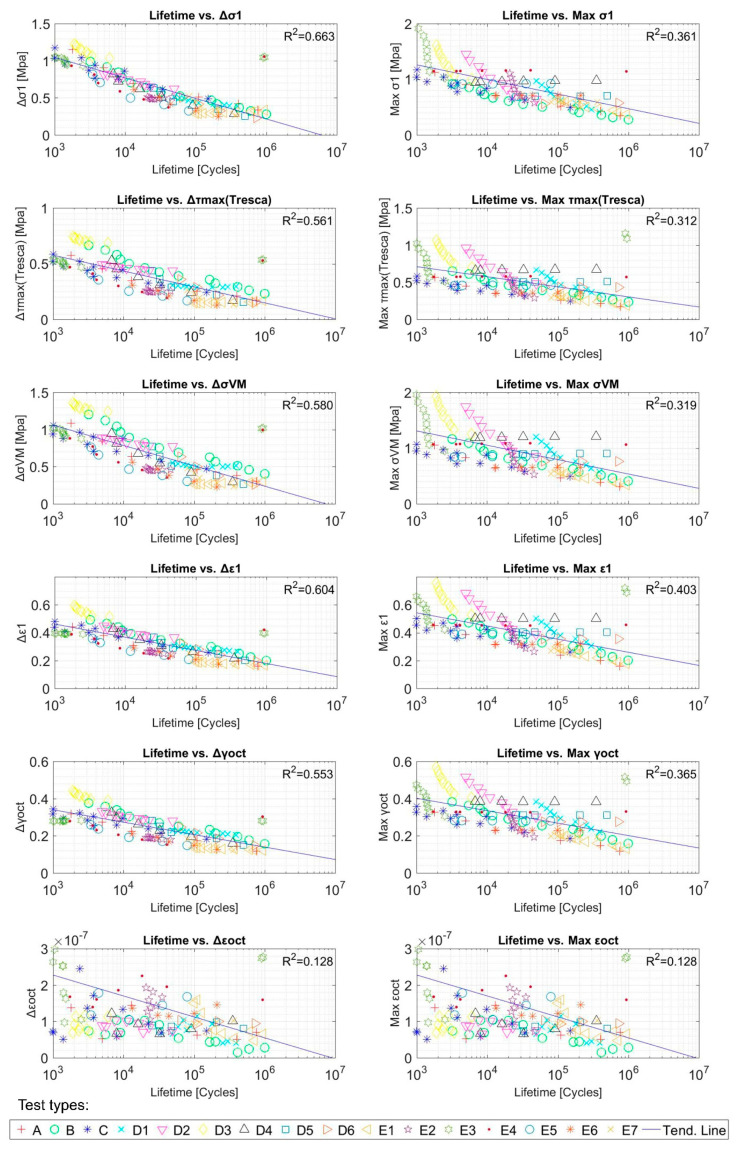

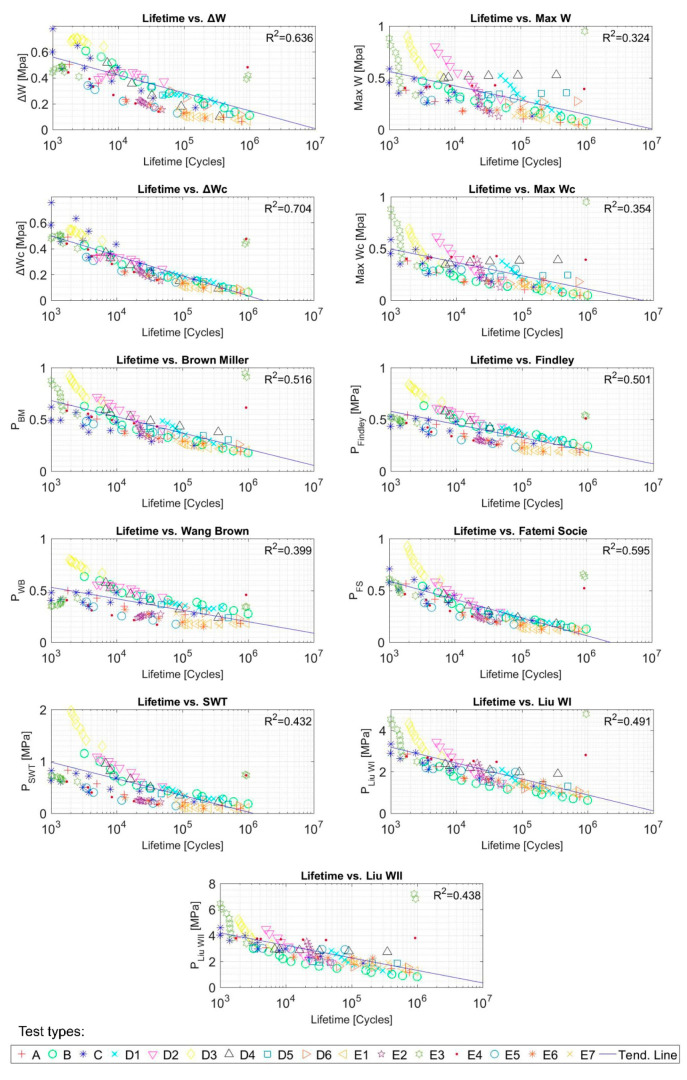
Fatigue parameters and tests lifetime (cycles) correlation for SBR tests. Initial data from [46].

Figure 11 represents the correlation between all of studied mechanical variables versus the lifetime of the samples. In order to differentiate each type of test (see column 2 of Table 2) related by the authors with letters (A, B, …, E7), these ones have been represented in plots by colours as referred in the legend.

## 6. Application of the Proposed Fatigue Damage Multi-Parameter (FDMP)

Although the fatigue parameter proposed in this work can be used with ‘*n*’ mechanical variables, for the experimental data described in Section 3.3, the proposed FDMP parameter has been used with seven variables. These variables are easily obtained by means of a FEM simulation, and have been used in order to show that from simple data, precise fatigue results can be achieved. In addition, the fatigue life of any component is dependent on the triaxial state of load it experiences. The variables that define this state of load are the ones chosen in the application of the proposed model for NBR and SBR: principal stresses, principal strains, and SED.

### 6.1. NBR – FDMP Results

For Mars and Fatemi tests with NBR material [45], the proposed multi-parameter model has been used, limited to seven variables. These seven variables are specified in Table 3. They have been chosen since they define the multiaxial stress state and the strain state experienced by each of the specimens. Therefore, the principal stresses, principal strains and SED are taken into account.

**Figure 12:**
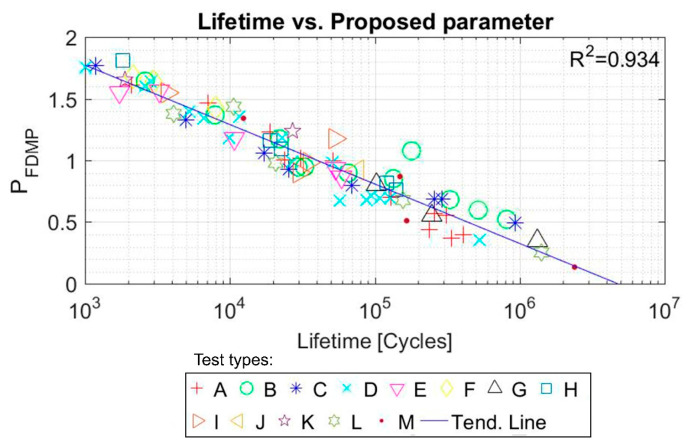
Proposed FDMP and tests lifetime (cycles) correlation for NBR tests. Initial data from [45].

The amplitude value, maximum value and minimum value obtained for each variable of each of the simulations are shown in Table S3, as supplementary material. After applying the GRG NonLinear regression algorithm in order [73,74] to maximize the value of the correlation coefficient *R*^2^, the values obtained for each of the weights are included in Table 3:

The parameters of the line for which the *R*^2^ value is maximized (Equation (11)) are A = −0.210, B = 3.200:

The values of the fatigue multiparameter for each test versus lifetime are plotted in Figure 12 below. The value of *R*^2^ is 0.934.

**Figure 13:**
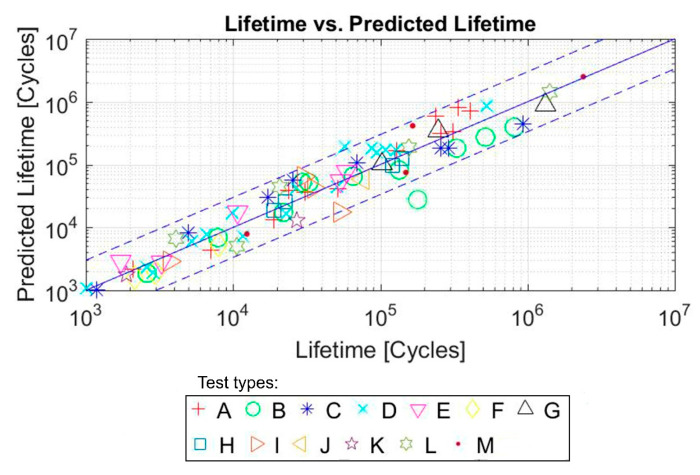
Predicted life with FDMP (cycles) versus real life (cycles) for NBR. Initial data from [45].

In order to check the accuracy of the model, a comparison was made between the real life and the expected lifetime of each of the test samples. As it can be seen in Figure 13, the continuous line would represent the predictions that coincide with the real life of the sample, and the dashed lines represent the real life multiplied and divided by 2.5, respectively. For this material, the proposed model is capable of including 78 out of the 83 Mars and Fatemi tests in these limits, which means 94% of the tests.

### 6.2. SBR–FDMP Results

For Ayoub tests with SBR material [46], the proposed multi-parameter model has been used, but just limited to the seven variables used for the NBR. These seven variables are specified in Table 4. They have been chosen since they define the multiaxial stress state and the strain state experienced by each of the specimens. Therefore, the principal stresses, principal strains, and SED are taken into account.

The amplitude value, maximum value, and minimum value obtained for each variable of each of the simulations are shown in Table S4, as supplementary material. After using the GRG nonlinear regression algorithm in order [73,74] to maximize the value of the correlation of the coefficient R^2^, the values obtained for each of the weights are included in Table 4.

**Figure 14:**
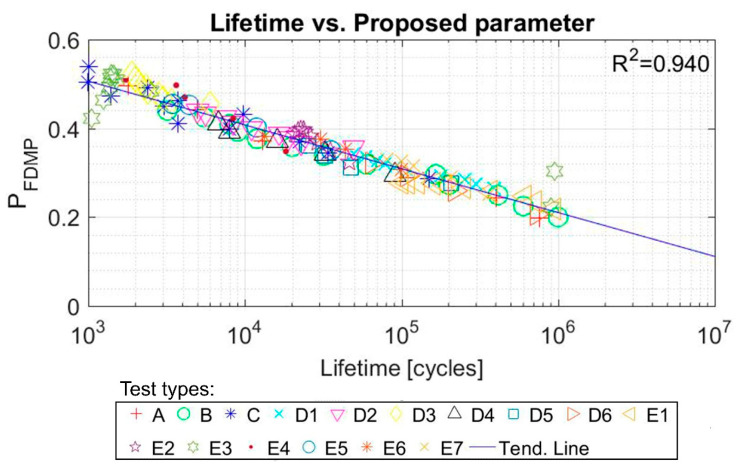
Proposed FDMP and tests lifetime (cycles) correlation for SBR tests. Initial data from [46].

The parameters of the line for which the *R*^2^ value is maximized (Equation (11)) are A = −0.043, B = 0.803. The values of the fatigue multiparameter for each test versus lifetime are plotted in Figure 14, obtaining an *R*^2^ value of 0.940.

**Figure 15:**
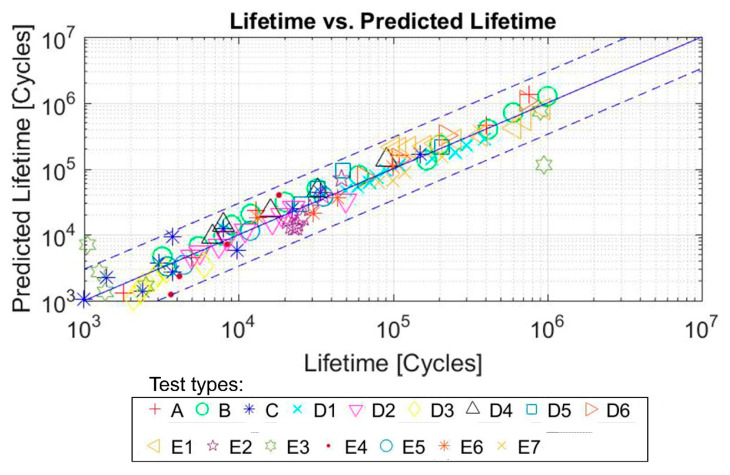
Predicted life (cycles) with FDMP versus real life (cycles) for SBR tests. Initial data from [46].

In order to check the accuracy of the model, a comparison was made between the real life and the expected lifetime of each of the test samples. As it can be seen in Figure 15, the continuous line would represent the predictions that coincide with the real life of the sample and the dashed lines represent the real life multiplied and divided by 2.5, respectively. For this material, the proposed model is capable of including 123 out of the 128 tests in these limits, which means 96% of the tests.

## 7. Discussion

As explained in Section 2 and Section 3, there is not a general model available in the literature which can be applied to obtain the fatigue life prediction in every type of rubber material. The difficulty arises due to the complex mechanical behavior of rubber materials and the large number of parameters influencing their durability [75].

The fatigue life prediction for rubber materials requires, on the one hand, a precise characterization of stress, strains and strain energy fields of the entire component along the complete load history and, on the other hand, a considerable effort is needed to carry out the corresponding fatigue tests, defining a fatigue model capable of correlating the selected fatigue parameter with the lifetime of each of the samples tested [36,75,76].

Initially, damage parameters based on stresses were used, based upon the wide experience with metallic materials [32]. However, this type of parameters has been unable to provide an accurate prediction in general cases, due to the differences in terms of mechanical and fatigue behavior between metallic and rubber materials.

In recent years, different authors have carried out specific studies making significant progress. To this respect, special reference should be made to the works of Mars and Fatemi [45]; and Ayoub and collaborators [46], with a lengthy series of tests and the proposal of specific fatigue parameters. Other authors have proposed different fatigue parameters for rubber materials, based on stresses (Lu [47], Abraham et al. [48], André et al. [49,50], Wang et al. [52]), on strains (Woo et al. [53], Suryatal et al. [54]), strain energy (Greensmith [57], Roberts and Benzies [56]), and parameters based on the critical plane (Mars and Fatemi [59], Verron [61,62], Fatemi-Socie [65], Smith, Watson, and Topper [66], Liu [67], Findley [68], Brown and Miller [69], Wang-Brown [70], McDiarmid [72]).

Despite all the published studies, the different proposed models for the fatigue of rubber materials provide a life prediction only valid for each of the specific studied materials and for the type of specimen used for the experimental testing. Thus, at present, there is not an objective and independent parameter that is able to predict the fatigue behavior of elastomeric materials for general component geometries and stress states.

In this context, the present work proposes a new general multiparameter, which is capable of including all the relevant information concerning stresses, strains, and strain energies simultaneously; therefore, improving the fatigue life prediction in any type of component and load state.

**Figure 16:**
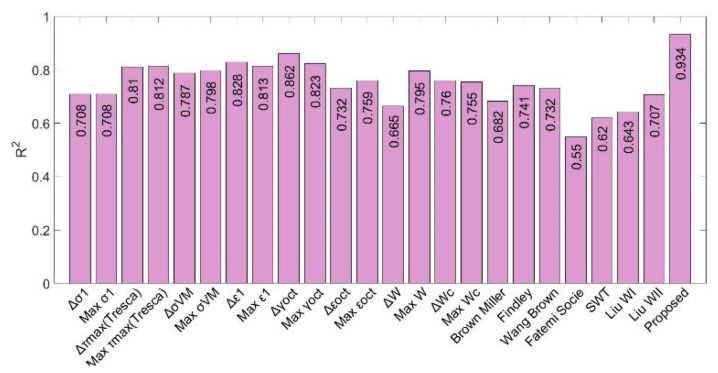
*R*^2^ correlation parameter for each fatigue parameter used for NBR tests.

**Figure 17:**
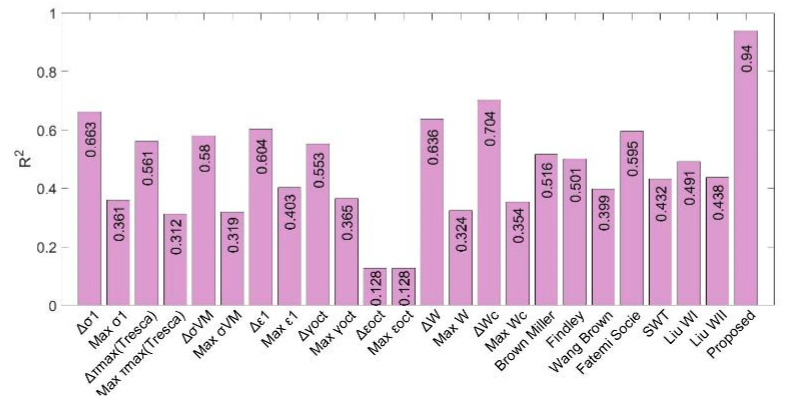
*R*^2^ correlation parameter for each fatigue parameter used for SBR tests.

The fatigue life predictions obtained by means of the proposed multiparameter have been compared with more than 20 different parameters used in the specialized literature, applied to the Mars and Fatemi tests [45] (Figure 16), and Ayoub tests [46] (Figure 17). As it can be seen in both figures, the value of the R^2^ coefficient (calculated by comparing the predicted values with the experimental ones) is considerably higher for the proposed multiparameter in both types of test.

The results obtained with the proposed FDMP model for tests from Mars and Fatemi [45] and Ayoub [46] show that to obtain good results in the prediction of fatigue life in complex states of load, it is efficient to take into account seven variables. However, these seven variables are not randomly selected variables. These variables have been chosen since they properly define the multiaxial stress and strain state experienced by each of the specimens. Thus, the stresses and strains in the three principal directions and also the strain energy density are taken into account. The results show that this state of stresses and strains together with the energy state effectively define the fatigue life for each of the specimens.

As a limitation of the present study, for a complete generalization of the applicability of the new proposed fatigue multiparameter, more results from experimental testing in different conditions and with different sample geometries would be needed. That would allow verifying the appropriate correlation between the predicted values and the experimental ones, fully validating the proposed model.

As the main limitation of the proposed FDMP model, it must be pointed out that a higher number of experimental results are needed in order to assure a correct adjustment by comparison with other simpler models; however, once the model has been adjusted for a specific material, their own parameters allow the identification of those most representative magnitudes for fatigue behavior, and then a more simplified model could be used in subsequent experimental testing for that material. It should be noted that, despite this limitation, the current model has been adjusted with the tests from Mars and Fatemi [45] and Ayoub [46], corresponding to different materials, demonstrating its reliability since it allows obtaining even more precise results than those obtained with the models of the authors themselves. Moreover, once the FDMP model has been programmed, it only needs the corresponding experimental data, requiring a minimum computational cost.

## 8. Conclusions

In this work, a new fatigue multiparameter for rubber material has been proposed. The underlying idea is to take into account different fatigue magnitudes, with their amplitude and mean values, assigning to each of them a pondered weight so that the sum of these values multiplied by their weights maximizes the correlation of the coefficient R^2^ with real experimental results.

Its application to different fatigue experimental testing results has proved the reliability of the new parameter for the fatigue life prediction in rubber materials.

In view of the obtained results, the proposed fatigue multiparameter could be considered as a promising improvement in the field of fatigue of rubber materials, helping to obtain better fatigue life predictions.

This work is only a first approach to a new fatigue multiparameter for rubber-like materials. Authors are working on the next steps for this parameter and its application for other collection data tests, type of samples, and different materials. This fatigue multiparameter will be a significant advance in the design of this rubber-like materials, as the lifetime of its components can be predicted. Additionally, the next future steps for the proposed parameter could be trying the unification of fatigue parameters for different materials.

The proposed FDMP model does not aim to be a universal and definitive approach for elastomers fatigue, but provide a reliable general tool that can be used for processing data obtained from experimental tests carried out under different conditions.

## Figures and Tables

**Table 1:** Information of Mars and Fatemi battery tests. Initial test data adapted from [45].

No	Test (Type-Number)	*δ*_max_ (mm)	*δ*_min_ (mm)	*θ*_max_ (°)	*θ*_min_ (°)	*P*_a_ (N)	*P*_m_ (N)	*T*_a_ (Nm)	*T*_m_ (Nm)	Offset (°)	Lifetime (Cycles)
1	A1	1.27	0	0	0	1136	581	0	1	0	339,167
83	I4	3.55	0	14	0	1645	638	60	47	180	12,408

**Table 2:** Information of the Ayoub battery tests. Initial test data adapted from [46].

Test	Test Type	F (Hz)	*d*_max_ (mm)	*d*_min_ (mm)	*θ*_max_ (°)	*θ*_min_ (°)	Lifetime (Cycles)
1	A	5	2.25	0	0	0	760,000
128	E7	5	3.8	1.75	0	0	363,500

**Table 3:** Model weights of FDMP model for NBR tests. Initial data from [45].

*n*	Variable	*γ*_n_	*α*_n_	*β*_n_
1	*σ*_1_	0.550	0.017	−0.188
2	*σ*_2_	0.932	−0.139	0.253
3	*σ*_3_	1.000	−0.068	−0.134
4	*ε*_1_	12.152	−0.153	2.580
5	*ε*_2_	1.000	1.500	−0.912
6	*ε*_3_	1.000	0.283	4.470
7	SED	−1.715	0.151	0.071

**Table 4:** Model weights of FDMP model for SBR tests. Initial data from [46].

*n*	Variable	*γ*_n_	*α*_n_	*β*_n_
1	*σ*_1_	0.006	319.264	−160.034
2	*σ*_2_	1.000	−1.371	0.962
3	*σ*_3_	1.000	−0.563	1.188
4	*ε*_1_	0.006	−25.018	13.890
5	*ε*_2_	1.000	−0.412	−0.297
6	*ε*_3_	1.000	−0.144	−1.452
7	SED	−0.093	16.546	−8115

## Supplementary Materials

The following are available online at https://www.mdpi.com/2073-4360/12/5/1194/s1, Tables S1–S4 are available as Supplementary Materials.

## Abbreviations

CED	Cracking Energy Density
FDMP	Fatigue Damage Multi-Parameter
FEM	Finite Element Method
HCF	High Cycle Fatigue
NBR	Acrylonitrile Butadiene Rubber
NR	Natural Rubber
P_BM_	Brown-Miller parameter
P_FS_	Fatemi-Socie parameter
P_WB_	Wang-Brown parameter
R^2^	Coefficient of determination
SBR	Styrene-Butadiene Rubber
SED	Strain Energy Density
SWT	Smith-Watson-Topper
