# Supplementary material for: A New Multiparameter Model for Multiaxial Fatigue Life Prediction of Rubber Materials
Source: Polymers (Basel). 2020 May 23;12(5):1194. doi: 10.3390/polym12051194 (PMC7285379; doi:10.3390/polym12051194)
Supplement: Supplementary file 1 [file polymers-12-01194-s001.pdf]

Table S1. Information of Mars and Fatemi battery tests. Initial test data adapted from [45].

No	Test (Type-Number)	δ_{max} (mm)	δ_{min} (mm)	θ_{max} (°)	θ_{min} (°)	P_a (N)	P_m (N)	T_a (Nm)	T_m (Nm)	Offset (°)	Lifetime (cycles)
1	A1	1.27	0.00	0.0	0.0	1,136	581	0	1	0	339,167
2	A2	1.50	0.02	0.5	−0.5	1,091	618	6	0	0	407,784
3	A3	1.52	0.00	0.0	0.0	1,220	567	0	0	0	238,547
4	A4	2.00	0.00	0.0	0.0	0	0	0	0	0	256,365
5	A5	2.03	0.01	0.5	−0.5	1,327	866	6	−1	0	311,987
6	A6	2.54	0.00	0.5	−0.5	1,540	766	5	−1	0	129,897
7	A7	3.80	0.00	0.2	−0.2	1,607	1,236	2	0	0	51,595
8	A8	3.81	0.00	0.5	−0.5	1,734	1,063	5	−1	0	30,775
9	A9	3.81	0.00	0.0	0.0	1,641	890	0	−1	0	23,773
10	A10	4.99	0.02	0.3	−0.3	1,866	1,295	2	−1	0	18,820
11	A11	6.36	0.00	0.3	−0.3	2,112	1,500	2	0	0	7,057
12	A12	7.44	0.00	0.0	0.0	2,727	1,756	0	1	0	2,082
13	A13	9.90	0.01	0.0	0.0	3,078	2,358	0	0	0	651
14	B1	0.25	−0.25	6.0	0.0	397	−204	31	21	0	813,883
15	B2	0.25	−0.25	7.0	0.0	444	−77	35	25	0	517,720
16	B3	0.01	0.00	7.7	0.0	75	−75	36	27	0	329,693
17	B4	0.01	−0.01	10.0	0.0	58	−235	45	34	0	65,536
18	B5	0.25	−0.25	10.0	0.0	394	−124	46	35	0	134,739
19	B6	0.25	−0.25	12.0	0.0	349	−314	51	40	0	32,768
20	B7	0.00	−0.03	12.0	0.0	24	−559	56	41	0	179,417
21	B8	0.25	−0.25	12.0	0.0	361	−176	54	43	0	29,454
22	B9	0.11	−0.10	15.1	0.0	147	−101	61	50	0	21,984
23	B10	0.04	0.01	15.4	0.0	80	−173	67	59	0	7,906
24	B11	0.10	−0.12	20.3	0.0	263	42	91	85	0	2,605
25	B12	0.04	−0.02	23.2	0.0	189	−142	114	96	0	924
26	C1	0.03	−0.03	5.4	−5.4	74	−233	54	1	0	928,635
27	C2	0.25	−0.25	7.0	−7.0	402	−253	61	0	0	291,426
28	C3	0.25	−0.25	7.0	−7.0	508	−370	67	0	0	256,648
29	C4	0.25	−0.25	7.9	−7.9	444	−205	68	−1	0	69,713
30	C5	0.01	0.00	9.0	−9.0	42	−299	76	−1	0	25,646
31	C6	0.25	−0.25	10.0	−10.0	353	−244	83	0	0	17,260
32	C7	0.25	−0.25	12.0	−12.0	356	−267	103	0	0	4,940
33	C8	0.25	−0.25	15.0	−15.0	425	−305	127	−1	0	956
34	C9	0.00	0.00	15.0	−15.0	74	−269	125	1	0	1,186
35	M1	0.20	0.19	7.0	−7.0	75	21	54	0	0	108,264
36	M2	0.08	0.07	7.0	−7.0	68	9	57	10	0	57,406
37	M3	0.43	0.38	12.0	−12.0	109	−1	103	0	0	11,689
38	M4	0.28	0.23	15.0	−15.0	102	15	123	1	0	1,005
39	J1	−1.43	−1.47	9.0	−9.0	48	−1,763	74	0	0	127,020
40	J2	−1.20	−1.26	14.6	−14.8	92	−1,779	130	−3	0	2,848
41	K1	2.82	2.76	8.0	−8.0	93	1,777	65	0	0	50,973
42	K2	3.14	3.09	12.0	−12.0	197	1,779	94	0	0	6,615
43	D1	2.04	0.03	4.0	0.0	1,288	836	20	16	0	87,538
44	D2	3.03	0.03	6.0	0.0	1,613	1,030	28	22	0	27,100
45	D3	3.76	0.01	7.1	0.0	1,641	1,063	26	23	0	23,024
46	D4	3.79	0.02	7.0	0.0	1,688	1,190	28	22	0	9,881
47	D5	4.55	0.02	13.6	0.0	2,067	1,506	59	11	0	2,599

No	Test (Type-Number)	δ_{max} (mm)	δ_{min} (mm)	θ_{max} (°)	θ_{min} (°)	P_a (N)	P_m (N)	T_a (Nm)	T_m (Nm)	Offset (°)	Lifetime (cycles)
48	D6	5.01	0.00	7.1	0.0	1,908	1,403	26	24	0	5,218
49	D7	0.76	0.00	3.0	0.0	681	337	19	26	0	524,288
50	D8	1.52	0.01	6.0	0.0	1,053	602	30	29	0	95,159
51	D9	2.01	0.03	7.0	0.0	1,043	783	28	35	0	58,722
52	D10	2.01	0.03	8.0	0.0	1,150	662	36	52	0	53,968
53	D11	2.54	0.00	10.0	0.0	1,371	927	43	52	0	10,693
54	D12	3.56	0.00	14.0	0.0	1,805	1,256	61	56	0	3,274
55	D13	3.56	−0.01	14.0	0.0	1,728	1,160	61	50	0	1,724
56	D14	3.80	0.01	15.0	0.0	1,874	1,326	65	76	0	2,990
57	D15	2.00	0.00	15.1	0.0	1,041	748	59	24	0	7,950
58	D16	2.03	0.01	20.0	0.0	1,168	751	86	52	0	2,149
59	E1	1.52	−1.52	3.0	−3.0	2,679	−742	33	−2	0	247,440
60	E2	2.03	−2.03	4.0	−4.0	3,711	−1,562	42	−2	0	102,226
61	E3	0.75	−0.74	3.0	−3.0	1,375	−295	35	−2	0	1,330,469
62	E4	1.52	−1.52	6.0	−6.0	2,589	−845	60	−2	0	120,890
63	E5	2.03	−2.03	8.0	−8.0	3,223	−1,233	72	−3	0	18,916
64	F1	−0.01	−1.51	12.0	0.0	1,253	−1,071	53	38	0	140,924
65	F2	0.00	−1.51	16.0	0.0	1,308	−1,397	72	55	0	22,614
66	F3	0.00	−1.52	20.0	0.0	1,296	−1,376	95	78	0	1,818
67	L1	0.00	−0.76	10.0	0.0	654	−618	45	32	180	35,070
68	L2	0.00	−1.52	10.0	0.0	1,461	−1,296	48	29	180	53,089
69	L3	−0.01	−1.52	12.0	0.0	1,414	−1,236	54	38	18	30,031
70	L4	−0.01	−1.52	14.0	0.0	1,370	−1,149	64	48	180	3,693
71	L5	0.00	−1.52	20.0	0.0	1,321	−896	103	86	180	76
72	G1	2.01	0.02	7.0	0.0	1,204	847	31	24	45	76,553
73	G2	2.53	0.00	10.0	0.0	1,379	888	43	35	45	27,078
74	G3	3.58	−0.02	14.1	0.0	1,795	1,272	59	51	45	1,887
75	H1	0.76	0.00	3.0	0.0	720	432	19	11	90	1,413,668
76	H2	2.01	0.01	7.0	0.0	1,158	621	31	24	90	156,381
77	H3	2.52	0.02	10.0	0.0	1,256	746	42	33	90	20,750
78	H4	3.53	0.00	14.0	0.0	1,586	886	58	47	90	4,096
79	H5	3.52	−0.11	14.1	0.0	1,608	874	57	46	90	10,602
80	I1	0.76	0.00	3.0	0.0	808	194	20	15	180	2,391,641
81	I2	2.03	0.00	7.0	0.0	1,219	646	32	24	180	166,014
82	I3	2.54	0.00	10.0	0.0	1,439	653	45	33	180	149,225
83	I4	3.55	0.00	14.0	0.0	1,645	638	60	47	180	12,408

Table S2. Information of Ayoub battery tests. Initial test data adapted from [46]

Test	Test type	F (Hz)	d_{max} (mm)	d_{min} (mm)	θ_{max} (°)	θ_{min} (°)	Lifetime (cycles)
1	A	5	2.25	0	0	0	760,000
2	A	5	2.81	0	0	0	402,500
3	A	5	3.38	0	0	0	110,500
4	A	5	4.5	0	0	0	13,000
5	A	5	5.63	0	0	0	5,000
6	A	5	6.75	0	0	0	1,800
7	A	5	7.88	0	0	0	600
8	A	5	9	0	0	0	200
9	B	3	0	0	40	0	1,000,000
10	B	3	0	0	45	0	600,000
11	B	3	0	0	50	0	413,510
12	B	3	0	0	55	0	200,000
13	B	3	0	0	60	0	165,654
14	B	3	0	0	65	0	60,000
15	B	3	0	0	70	0	32,000
16	B	3	0	0	75	0	20,000
17	B	3	0	0	80	0	12,000
18	B	3	0	0	85	0	9,000
19	B	3	0	0	90	0	8,000
20	B	3	0	0	95	0	5,500
21	B	3	0	0	100	0	3,201
22	C	5	2.25	0	40	0	150,000
23	C	5	3.38	0	40	0	33,725
24	C	5	4.5	0	40	0	3,750
25	C	5	5.63	0	40	0	1,385
26	C	5	2.25	0	60	0	22,365
27	C	5	3.38	0	60	0	8,000
28	C	5	4.5	0	60	0	3,036
29	C	5	5.63	0	60	0	1,000
30	C	5	2.25	0	80	0	9,785
31	C	5	3.375	0	80	0	3,750
32	C	5	4.5	0	80	0	2,379
33	C	5	5.63	0	80	0	1,009
34	D1	5	0	0	55	5	392,612
35	D1	5	0	0	60	10	300,000
36	D1	5	0	0	65	15	255,000
37	D1	5	0	0	70	20	180,000
38	D1	5	0	0	75	25	110,000
39	D1	5	0	0	80	30	89,200
40	D1	5	0	0	85	35	78,900
41	D1	5	0	0	90	40	69,900
42	D1	5	0	0	95	45	58,960
43	D1	5	0	0	100	50	49,586
44	D2	5	0	0	77	7	49,565
45	D2	5	0	0	84	14	22,749
46	D2	5	0	0	91	21	19,000
47	D2	5	0	0	98	28	16,561

Test	Test type	F (Hz)	d_{max} (mm)	d_{min} (mm)	θ_{max} (°)	θ_{min} (°)	Lifetime (cycles)
48	D2	5	0	0	105	35	11,000
49	D2	5	0	0	112	42	8,436
50	D2	5	0	0	119	49	7,500
51	D2	5	0	0	126	56	5,579
52	D2	5	0	0	133	63	5,000
53	D3	5	0	0	105	5	6,000
54	D3	5	0	0	110	10	3,415
55	D3	5	0	0	115	15	3,000
56	D3	5	0	0	120	20	2,800
57	D3	5	0	0	125	25	2,400
58	D3	5	0	0	130	30	2,315
59	D3	5	0	0	135	35	2,100
60	D3	5	0	0	140	40	2,015
61	D3	5	0	0	145	45	1,900
62	D4	5	0	0	100	20	6,811
63	D4	5	0	0	100	30	8,010
64	D4	5	0	0	100	40	16,210
65	D4	5	0	0	100	50	32,664
66	D4	5	0	0	100	60	90,500
67	D4	5	0	0	100	70	350,000
68	D5	5	0	0	80	10	25,411
69	D5	5	0	0	80	20	32,594
70	D5	5	0	0	80	30	47,715
71	D5	5	0	0	80	40	208,700
72	D5	5	0	0	80	50	502,400
73	D6	5	0	0	70	10	64,503
74	D6	5	0	0	70	20	108,875
75	D6	5	0	0	70	30	220,000
76	D6	5	0	0	70	40	725,618
77	E1	5	2.45	0.2	0	0	941,596
78	E1	5	2.65	0.4	0	0	710,654
79	E1	5	2.85	0.6	0	0	615,562
80	E1	5	3.05	0.8	0	0	370,123
81	E1	5	3.25	1	0	0	250,116
82	E1	5	3.45	1.2	0	0	190,120
83	E1	5	3.65	1.4	0	0	160,150
84	E1	5	3.85	1.6	0	0	128,250
85	E1	5	4.05	1.8	0	0	110,000
86	E1	5	4.25	2	0	0	100,000
87	E2	5	3.73	0.35	0	0	46,422
88	E2	5	4.08	0.7	0	0	35,200
89	E2	5	4.43	1.05	0	0	29,023
90	E2	5	4.78	1.4	0	0	26,120
91	E2	5	5.13	1.75	0	0	24,048
92	E2	5	5.48	2.1	0	0	23,700
93	E2	5	5.83	2.45	0	0	22,752
94	E2	5	6.18	2.8	0	0	21,900
95	E2	5	6.53	3.15	0	0	20,800

Test	Test type	F (Hz)	d_{max} (mm)	d_{min} (mm)	θ_{max} (°)	θ_{min} (°)	Lifetime (cycles)
96	E3	5	6.18	0.55	0	0	2,512
97	E3	5	6.73	1.1	0	0	1,511
98	E3	5	7.28	1.65	0	0	1,450
99	E3	5	7.83	2.2	0	0	1,405
100	E3	5	8.38	2.75	0	0	1,390
101	E3	5	8.93	3.3	0	0	1,367
102	E3	5	9.48	3.85	0	0	1,250
103	E3	5	10.03	4.4	0	0	1,050
104	E3	5	10.58	4.95	0	0	1,000
105	E3	5	11.13	5.5	0	0	950,000
106	E3	5	11.68	6.05	0	0	901,000
107	E4	5	6.75	0.6	0	0	934,000
108	E4	5	6.75	1.2	0	0	1,735
109	E4	5	6.75	1.8	0	0	3,647
110	E4	5	6.75	2.4	0	0	4,141
111	E4	5	6.75	3	0	0	8,422
112	E4	5	6.75	3.6	0	0	18,284
113	E4	5	6.75	4.2	0	0	40,973
114	E5	5	5.63	0.55	0	0	3,444
115	E5	5	5.63	1.1	0	0	4,414
116	E5	5	5.63	2.2	0	0	11,887
117	E5	5	5.63	2.7	0	0	35,484
118	E5	5	5.63	3.2	0	0	79,455
119	E6	5	4.5	0.5	0	0	13,321
120	E6	5	4.5	1	0	0	30,501
121	E6	5	4.5	1.5	0	0	43,626
122	E6	5	4.5	2	0	0	100,426
123	E6	5	4.5	2.5	0	0	209,037
124	E7	5	3.8	0.35	0	0	80,000
125	E7	5	3.8	0.7	0	0	100,200
126	E7	5	3.8	1.05	0	0	120,000
127	E7	5	3.8	1.4	0	0	206,713
128	E7	5	3.8	1.75	0	0	363,500

Table S3. Values of var_amp. var_max and var_min for each variable of FDMP model for NBR tests. Initial test data adapted from [45].

	σ_1 (MPa)			σ_2 (MPa)			σ_3 (MPa)		
**Test**	σ_{amp}	σ_{max}	σ_{min}	σ_{amp}	σ_{max}	σ_{min}	σ_{amp}	σ_{max}	σ_{min}
A1	1.39284	2.80039	0.01472	0.51357	1.06963	0.04248	0.01268	0.02670	0.00134
A2	1.54733	3.26293	0.16826	0.57087	1.19423	0.05249	0.07973	0.08293	−0.07654
A3	1.66173	3.34071	0.01725	0.58283	1.22268	0.05701	0.01405	0.02997	0.00188
A4	2.17979	4.37932	0.01974	0.69524	1.47809	0.08761	0.01582	0.03471	0.00306
A5	2.12613	4.40303	0.15078	0.69683	1.47583	0.08217	0.08984	0.09587	−0.08382
A6	2.69046	5.50992	0.12899	0.79229	1.69782	0.11325	0.09897	0.10672	−0.09122
A7	4.15535	8.34361	0.03290	0.95710	2.12121	0.20701	0.05030	0.07115	−0.02945
A8	4.11987	8.34014	0.10040	0.95636	2.11098	0.19825	0.09911	0.11392	−0.08431
A9	4.19999	8.39191	−0.00807	0.95842	2.12788	0.21104	0.01688	0.04092	0.00716
A10	5.53415	11.10746	0.03916	1.05305	2.39139	0.28529	0.05033	0.07211	−0.02855
A11	7.12365	14.30277	0.05547	1.12023	2.60760	0.36713	0.01590	0.04193	0.01014
A12	8.56802	17.07698	−0.05906	1.12780	2.71806	0.46246	0.01880	0.04657	0.00896
A13	11.40100	22.72341	−0.07859	1.50071	3.61678	0.61536	0.02502	0.06196	0.01192
B1	1.62187	2.98641	−0.25733	0.14443	0.09332	−0.19554	0.28372	−0.56273	−1.13016
B2	1.94064	3.57697	−0.30432	0.14338	0.09453	−0.19222	0.36805	−0.58067	−1.31677
B3	1.95194	3.72734	−0.17654	0.00568	0.00154	−0.00982	0.87507	−0.09758	−1.84771
B4	2.83086	5.33647	−0.32526	0.00977	0.00192	−0.01762	1.01579	−0.16441	−2.19598
B5	3.06445	5.63484	−0.49407	0.14178	0.09850	−0.18507	0.57350	−0.64164	−1.78864
B6	3.96142	7.26381	−0.65903	0.14134	0.09586	−0.18683	0.68013	−0.68488	−2.04514
B7	3.71390	6.92583	−0.50197	0.01595	0.00316	−0.02875	1.10867	−0.24584	−2.46317
B8	3.96142	7.26381	−0.65903	0.14134	0.09586	−0.18683	0.68013	−0.68488	−2.04514
B9	5.45732	10.03148	−0.88315	0.05418	0.03040	−0.07797	1.08809	−0.44438	−2.62056
B10	5.54847	10.28014	−0.81681	0.02996	0.01313	−0.04678	1.26401	−0.24615	−2.77418
B11	8.71807	15.93054	−1.50559	0.04675	0.00569	−0.08781	1.23510	−0.60000	−3.07019
B12	10.88640	19.89193	−1.88088	0.06217	0.01753	−0.10682	1.45593	−0.47227	−3.38413
C1	1.23976	2.62264	0.14312	0.00332	0.00063	−0.00600	0.71403	−0.17175	−1.59981
C2	1.93703	3.85096	−0.02311	0.01003	0.00528	−0.01479	1.02802	−0.06040	−2.11645
C3	1.93703	3.85096	−0.02311	0.01003	0.00528	−0.01479	1.02802	−0.06040	−2.11645
C4	2.24725	4.48817	−0.00632	0.01156	0.00550	−0.01762	1.09432	−0.09292	−2.28157
C5	2.42400	5.10449	0.25650	0.01080	0.00384	−0.01776	0.97267	−0.31179	−2.25713
C6	3.06084	6.14691	0.02523	0.01596	0.00617	−0.02575	1.22836	−0.16481	−2.62153
C7	3.95781	7.96063	0.04501	0.02169	0.00739	−0.03600	1.33495	−0.22752	−2.89742
C8	5.53714	11.13006	0.05578	0.03284	0.00983	−0.05585	1.46819	−0.31059	−3.24698
C9	5.28753	10.88352	0.30846	0.02432	0.00182	−0.04683	1.27673	−0.49423	−3.04770
M1	1.67054	3.94853	0.60746	0.10242	0.12840	−0.07643	0.69275	−0.13020	−1.51571
M2	1.69462	3.74944	0.36021	0.04350	0.04864	−0.03836	0.77765	−0.19831	−1.75362
M3	3.67558	8.45406	1.10290	0.20576	0.25609	−0.15542	0.89106	−0.16936	−1.95148
M4	5.27896	11.37138	0.81346	0.14768	0.16274	−0.13263	1.12120	−0.32616	−2.56856
J1	0.81685	1.22950	−0.40420	1.11967	0.25943	−1.97991	0.61017	−3.83220	−5.05255
J2	3.69828	6.84142	−0.55513	0.88192	0.43202	−1.33182	0.96381	−3.29021	−5.21784
K1	1.64208	9.32408	6.03993	0.46568	1.64432	0.71296	0.00127	0.03388	0.03134
K2	3.29435	13.34786	6.75917	0.68486	1.67791	0.30819	0.00291	0.03404	0.02823
D1	2.69562	5.42006	0.02881	0.46976	1.04214	0.10262	0.01509	0.03410	0.00392
D2	4.21814	8.37917	−0.05712	0.56256	1.27921	0.15408	0.01590	0.03829	0.00649
D3	5.34916	10.52051	−0.17780	0.63835	1.45264	0.17594	0.01621	0.03960	0.00718

D4	5.33840	10.52491	−0.15189	0.64456	1.47814	0.18903	0.01609	0.03963	0.00744
D5	8.81317	17.06139	−0.56496	0.37333	0.91823	0.17158	0.01466	0.03711	0.00779
D6	6.77025	13.27736	−0.26315	0.81816	1.88849	0.25216	0.01655	0.04088	0.00778
D7	1.27636	2.53314	−0.01959	0.07240	0.14792	0.00312	0.00827	0.01714	0.00059
D8	2.75834	5.43263	−0.08404	0.11928	0.26090	0.02235	0.01259	0.02748	0.00229
D9	3.51548	6.94109	−0.08987	0.20650	0.47831	0.06531	0.01406	0.03205	0.00393
D10	3.86408	7.58366	−0.14451	0.12818	0.30646	0.05010	0.01360	0.03118	0.00398
D11	5.19581	10.04158	−0.35004	0.17095	0.36891	0.02700	0.01442	0.03351	0.00467
D12	8.16943	15.58116	−0.75770	0.19486	0.43562	0.04590	0.01439	0.03476	0.00598
D13	8.16957	15.57279	−0.76635	0.20029	0.43587	0.03529	0.02581	0.04501	−0.00660
D14	8.97493	17.08627	−0.86359	0.18995	0.43979	0.05988	0.01427	0.03465	0.00610
D15	7.24908	13.66594	−0.83221	0.00866	0.02072	0.00340	0.27838	−0.11573	−0.67250
D16	10.26607	19.24451	−1.28763	0.00615	0.01219	−0.00011	0.44143	−0.21481	−1.09766
E1	2.02751	3.21739	−0.83763	1.35824	1.10936	−1.60711	2.15310	0.91177	−3.39443
E2	2.77368	4.37745	−1.16990	2.05834	1.57448	−2.54221	3.12131	1.37177	−4.87085
E3	1.27994	2.03304	−0.52685	0.14879	0.14606	−0.15152	1.14098	0.46275	−1.81920
E4	2.80784	4.41318	−1.20250	0.41175	0.34358	−0.47993	2.37735	0.98199	−3.77270
E5	4.01372	6.24243	−1.78502	0.74166	0.57253	−0.91078	3.41394	1.47537	−5.35251
F1	3.72665	6.23078	−1.22252	1.21722	0.51996	−1.91447	0.82645	−2.12998	−3.78287
F2	5.89975	10.05702	−1.74249	1.21041	0.55289	−1.86794	0.66094	−2.51214	−3.83402
F3	8.40333	14.52845	−2.27821	1.19768	0.58184	−1.81351	0.50717	−2.86483	−3.87918
L1	2.02593	3.76584	−0.28603	0.03023	0.00676	−0.05369	1.69188	−0.12681	−3.51057
L2	1.02252	1.89348	−0.15155	0.06594	0.01903	−0.11285	2.62703	−0.02045	−5.27451
L3	3.68999	6.23769	−1.14229	1.22875	0.52711	−1.93038	0.87675	−2.10145	−3.85495
L4	2.83594	5.16419	−0.50770	0.10914	0.03714	−0.18115	2.84373	−0.08860	−5.77606
L5	6.57059	11.81587	−1.32532	0.20615	0.08074	−0.33155	3.17205	−0.10809	−6.45220
G1	3.25176	6.76804	0.26452	0.43830	0.77207	−0.10454	0.28214	0.17214	−0.39214
G2	4.72468	9.70282	0.25345	0.46986	0.80258	−0.13714	0.39269	0.20668	−0.57870
G3	7.52825	15.21487	0.15836	0.57631	0.97916	−0.17345	0.51073	0.27973	−0.74173
H1	0.90470	2.23336	0.42395	0.29406	0.48915	−0.09897	0.30598	0.13404	−0.47793
H2	2.50466	6.10020	1.09087	0.61600	1.05549	−0.17651	0.58030	0.28779	−0.87281
H3	3.63243	8.73501	1.47014	0.68993	1.16080	−0.21906	0.74072	0.34743	−1.13401
H4	5.83292	13.60474	1.93890	0.82240	1.38161	−0.26319	0.91225	0.43675	−1.38776
H5	5.92997	13.63210	1.77216	0.82006	1.36255	−0.27757	0.98903	0.46225	−1.51580
I1	0.24926	1.57723	1.07872	0.34495	0.59620	−0.09371	0.44179	0.20203	−0.68155
I2	0.59732	4.16793	2.97329	0.70630	1.25718	−0.15541	0.84488	0.40938	−1.28039
I3	0.58563	5.59392	4.42266	0.80583	1.40678	−0.20489	1.05131	0.48419	−1.61843
I4	1.51510	9.09826	6.06807	0.95235	1.66161	−0.24309	1.25203	0.58268	−1.92137

	ε_1			ε_2			ε_3		
**Test**	ε_{amp}	ε_{max}	ε_{min}	ε_{amp}	ε_{max}	ε_{min}	ε_{amp}	ε_{max}	ε_{min}
A1	0.12628	0.25198	−0.00059	0.00435	−0.00020	−0.00889	0.09729	−0.00508	−0.19966
A2	0.13593	0.29256	0.02070	0.00455	−0.00397	−0.01307	0.10040	−0.02151	−0.22230
A3	0.15178	0.30278	−0.00077	0.00505	−0.00027	−0.01037	0.11249	−0.00696	−0.23193
A4	0.20088	0.40073	−0.00103	0.00627	−0.00044	−0.01298	0.13873	−0.01110	−0.28856
A5	0.19029	0.39947	0.01889	0.00620	−0.00337	−0.01576	0.12946	−0.02517	−0.28409
A6	0.24263	0.50262	0.01736	0.00807	−0.00250	−0.01863	0.15355	−0.02937	−0.33647
A7	0.37706	0.76116	0.00704	0.00978	−0.00181	−0.02137	0.20547	−0.03624	−0.44719
A8	0.36997	0.75823	0.01830	0.01010	−0.00338	−0.02358	0.20169	−0.04147	−0.44486
A9	0.38361	0.76752	0.00030	0.00974	−0.00126	−0.02073	0.21139	−0.03023	−0.45300

A10	0.48913	0.99461	0.01636	0.00958	−0.00474	−0.02391	0.23622	−0.05284	−0.52528
A11	0.60862	1.24610	0.02886	0.00998	−0.00667	−0.02664	0.26460	−0.06735	−0.59655
A12	0.71088	1.45044	0.02868	0.01327	−0.00346	−0.02999	0.28153	−0.07949	−0.64255
A13	0.94593	1.93002	0.03817	0.01765	−0.00460	−0.03991	0.37462	−0.10577	−0.85501
B1	0.18762	0.38839	0.01315	0.03441	0.02846	−0.04037	0.10782	−0.04515	−0.26079
B2	0.22475	0.45994	0.01044	0.03386	0.02887	−0.03885	0.12641	−0.04757	−0.30038
B3	0.26051	0.51088	−0.01014	0.00215	0.00347	−0.00083	0.17258	−0.01158	−0.35673
B4	0.35206	0.68827	−0.01585	0.00404	0.00770	−0.00038	0.20822	−0.01989	−0.43634
B5	0.34215	0.68643	0.00212	0.03689	0.03513	−0.03866	0.17562	−0.05586	−0.40709
B6	0.42377	0.84591	−0.00164	0.03503	0.03490	−0.03515	0.20334	−0.06221	−0.46888
B7	0.43459	0.84837	−0.02081	0.00701	0.01489	0.00087	0.23403	−0.02960	−0.49766
B8	0.42377	0.84591	−0.00164	0.03503	0.03490	−0.03515	0.20334	−0.06221	−0.46888
B9	0.56984	1.11358	−0.02610	0.00715	0.01321	−0.00109	0.25871	−0.05091	−0.56832
B10	0.58923	1.14600	−0.03246	0.00862	0.01263	−0.00462	0.27296	−0.03817	−0.58408
B11	0.80027	1.56836	−0.03218	0.00679	0.02139	0.00780	0.29830	−0.07883	−0.67543
B12	0.94262	1.84262	−0.04263	0.01777	0.03404	−0.00150	0.32523	−0.07981	−0.73027
C1	0.17510	0.37973	0.02952	0.00557	0.00699	−0.00414	0.13141	−0.03128	−0.29409
C2	0.24395	0.51750	0.02960	0.04662	0.04972	−0.04353	0.17190	−0.03157	−0.37537
C3	0.24395	0.51750	0.02960	0.04662	0.04972	−0.04353	0.17190	−0.03157	−0.37537
C4	0.27842	0.59171	0.03488	0.04678	0.05052	−0.04304	0.18747	−0.03729	−0.41223
C5	0.31159	0.67466	0.05147	0.00457	0.00765	−0.00149	0.19396	−0.05502	−0.44294
C6	0.36238	0.77210	0.04734	0.04721	0.05275	−0.04167	0.22012	−0.05063	−0.49087
C7	0.44644	0.95236	0.05949	0.04768	0.05529	−0.04007	0.24692	−0.06317	−0.55701
C8	0.57873	1.23588	0.07842	0.04848	0.05978	−0.03719	0.28051	−0.08141	−0.64243
C9	0.57072	1.22741	0.08597	0.01087	0.02141	−0.00033	0.27127	−0.09062	−0.63316
M1	0.22285	0.51571	0.07001	0.01658	−0.01188	−0.04503	0.13504	−0.05749	−0.32757
M2	0.22926	0.50954	0.05101	0.00654	−0.00355	−0.01663	0.15077	−0.04772	−0.34927
M3	0.41405	0.95917	0.13107	0.03359	−0.02207	−0.08925	0.18701	−0.10280	−0.47682
M4	0.55505	1.23461	0.12452	0.02127	−0.00982	−0.05237	0.24028	−0.10816	−0.58872
J1	0.10291	0.50506	0.29924	0.17407	0.41127	0.06312	0.13079	−0.28149	−0.54308
J2	0.39498	1.02740	0.23744	0.15612	0.40037	0.08813	0.20466	−0.27489	−0.68421
K1	0.15909	0.88015	0.56196	0.08422	−0.01969	−0.18812	0.00273	−0.34580	−0.35126
K2	0.28744	1.20915	0.63427	0.13081	−0.02919	−0.29080	0.00340	−0.36942	−0.37621
D1	0.25986	0.52234	0.00261	0.04409	−0.00311	−0.09129	0.13764	−0.01663	−0.29192
D2	0.39502	0.79088	0.00084	0.05961	−0.00588	−0.12510	0.18096	−0.02661	−0.38852
D3	0.48682	0.97055	−0.00309	0.06477	−0.00753	−0.13708	0.20818	−0.03135	−0.44771
D4	0.48563	0.97019	−0.00107	0.06317	−0.00730	−0.13363	0.20824	−0.03330	−0.44978
D5	0.73897	1.48000	0.00206	0.12721	−0.02330	−0.27772	0.22362	−0.04776	−0.49499
D6	0.59181	1.18207	−0.00156	0.05312	−0.00707	−0.11331	0.24368	−0.04370	−0.53107
D7	0.13483	0.26825	−0.00141	0.05018	−0.00201	−0.10237	0.06223	−0.00211	−0.12656
D8	0.28039	0.55717	−0.00361	0.08810	−0.00648	−0.18268	0.11057	−0.00887	−0.23001
D9	0.34582	0.68998	−0.00166	0.09280	−0.00795	−0.19355	0.13535	−0.01648	−0.28717
D10	0.37958	0.75566	−0.00350	0.10873	−0.01006	−0.22752	0.13500	−0.01653	−0.28653
D11	0.48887	0.96580	−0.01193	0.12552	−0.01484	−0.26588	0.16229	−0.01663	−0.34120
D12	0.70407	1.38960	−0.01854	0.15315	−0.02369	−0.32999	0.20043	−0.02765	−0.42851
D13	0.70315	1.38815	−0.01815	0.15319	−0.02388	−0.33026	0.19947	−0.02807	−0.42701
D14	0.75671	1.49495	−0.01846	0.15925	−0.02575	−0.34425	0.20707	−0.03196	−0.44611
D15	0.65828	1.29100	−0.02556	0.13354	−0.01184	−0.27891	0.20527	−0.03072	−0.44126
D16	0.85566	1.69128	−0.02005	0.12745	−0.01770	−0.27260	0.24669	−0.05413	−0.54752
E1	0.19393	0.41283	0.02496	0.09021	0.09923	−0.08119	0.16851	−0.00623	−0.34324

E2	0.26680	0.57205	0.03845	0.12267	0.13865	−0.10669	0.21813	−0.00683	−0.44310
E3	0.13366	0.25935	−0.00797	0.11727	0.12484	−0.10970	0.11727	0.00695	−0.22759
E4	0.28135	0.55289	−0.00982	0.24008	0.26994	−0.21022	0.21926	0.01168	−0.42685
E5	0.38383	0.76344	−0.00421	0.32833	0.38108	−0.27557	0.27588	0.01188	−0.53989
F1	0.32917	0.80783	0.14948	0.09121	0.17039	−0.01202	0.10712	−0.25327	−0.46751
F2	0.48244	1.11817	0.15329	0.10185	0.19213	−0.01157	0.14791	−0.26707	−0.56289
F3	0.65590	1.45588	0.14408	0.11709	0.22228	−0.01190	0.17864	−0.28274	−0.64002
L1	0.32826	0.63528	−0.02123	0.08593	0.16699	−0.00487	0.24233	−0.01890	−0.50357
L2	0.31121	0.59786	−0.02455	0.19041	0.36466	−0.01616	0.27670	−0.02145	−0.57484
L3	0.33243	0.81781	0.15294	0.10371	0.18412	−0.02330	0.12181	−0.24153	−0.48515
L4	0.48954	0.93843	−0.04066	0.20006	0.38181	−0.01830	0.31847	−0.03861	−0.67555
L5	0.78158	1.49552	−0.06763	0.21629	0.40888	−0.02371	0.36303	−0.06240	−0.78847
G1	0.31933	0.67848	0.03982	0.10169	0.01273	−0.19065	0.13042	−0.04435	−0.30518
G2	0.44837	0.94642	0.04968	0.13230	0.01731	−0.24730	0.14921	−0.06947	−0.36788
G3	0.65456	1.37669	0.06758	0.16409	0.01842	−0.30976	0.18229	−0.09675	−0.46133
H1	0.09712	0.24296	0.04872	0.04842	0.01724	−0.07960	0.05063	−0.04595	−0.14721
H2	0.24915	0.62587	0.12756	0.09868	0.03063	−0.16674	0.10805	−0.10160	−0.31771
H3	0.35465	0.87822	0.16891	0.12412	0.03556	−0.21267	0.13710	−0.12579	−0.39999
H4	0.51747	1.27104	0.23610	0.16021	0.04827	−0.27216	0.16894	−0.16329	−0.50117
H5	0.52496	1.27526	0.22535	0.16872	0.06222	−0.27521	0.18050	−0.15328	−0.51428
I1	0.02868	0.17624	0.11888	0.02733	0.00611	−0.04856	0.04463	−0.06435	−0.15360
I2	0.06927	0.45313	0.31459	0.05993	0.01324	−0.10662	0.08048	−0.15863	−0.31959
I3	0.13541	0.66717	0.39634	0.08155	0.02088	−0.14222	0.11307	−0.18604	−0.41218
I4	0.21737	0.98498	0.55024	0.10822	0.03002	−0.18642	0.13496	−0.24161	−0.51154

	**SED (MPa)**		
**Test**	W_{amp}	W_{max}	W_{min}
A1	0.15015	0.26405	−0.03625
A2	0.20460	0.36426	−0.04494
A3	0.20978	0.36961	−0.04995
A4	0.34642	0.61257	−0.08027
A5	0.35576	0.63250	−0.07903
A6	0.53035	0.94382	−0.11688
A7	1.05825	1.89505	−0.22146
A8	1.06282	1.90527	−0.22037
A9	1.06292	1.90316	−0.22268
A10	1.65339	2.98471	−0.32206
A11	2.38668	4.35342	−0.41993
A12	3.04803	5.57725	−0.51880
A13	4.05585	7.42135	−0.69034
B1	0.34040	0.59950	−0.08130
B2	0.46308	0.81340	−0.11277
B3	0.56322	0.98083	−0.14560
B4	0.94908	1.65302	−0.24514
B5	0.94373	1.65153	−0.23592
B6	1.35771	2.37349	−0.34193
B7	1.36560	2.37893	−0.35227
B8	1.35771	2.37349	−0.34193
B9	2.15321	3.75374	−0.55268

B10	2.24270	3.90766	−0.57775
B11	3.79019	6.65455	−0.92584
B12	4.94399	8.67890	−1.20908
C1	0.27754	0.54181	−0.01326
C2	0.47547	0.92571	−0.02522
C3	0.47547	0.92571	−0.02522
C4	0.60355	1.17516	−0.03194
C5	0.76925	1.50240	−0.03610
C6	0.96218	1.87371	−0.05064
C7	1.38120	2.69009	−0.07230
C8	2.15041	4.18930	−0.11151
C9	2.13013	4.16211	−0.09814
M1	0.46376	0.91349	−0.01403
M2	0.46488	0.90908	−0.02069
M3	1.35522	2.67948	−0.03095
M4	2.11830	4.15225	−0.08436
J1	0.82178	2.13290	0.48934
J2	2.17446	4.58286	0.23394
K1	0.59737	2.40592	1.21119
K2	1.32318	4.08736	1.44101
D1	0.50638	0.89512	−0.11764
D2	1.05011	1.86268	−0.23754
D3	1.49896	2.66373	−0.33420
D4	1.49994	2.66805	−0.33183
D5	3.04490	5.41770	−0.67210
D6	2.12188	3.79781	−0.44594
D7	0.14094	0.24614	−0.03573
D8	0.54323	0.95194	−0.13453
D9	0.80086	1.40842	−0.19330
D10	0.93884	1.64916	−0.22853
D11	1.44652	2.54010	−0.35293
D12	2.72954	4.80395	−0.65513
D13	2.72953	4.80245	−0.65661
D14	3.09920	5.45835	−0.74005
D15	2.43874	4.26433	−0.61314
D16	3.92375	6.90543	−0.94207
E1	0.38911	0.71136	−0.06686
E2	0.75644	1.34492	−0.16795
E3	0.15074	0.28885	−0.01263
E4	0.66150	1.23149	−0.09150
E5	1.25909	2.29330	−0.22487
F1	1.12832	2.35818	0.10154
F2	2.16381	4.18331	−0.14431
F3	3.41707	6.43128	−0.40285
L1	1.03766	1.79703	−0.27828
L2	1.30470	2.24611	−0.36329
L3	1.18842	2.44688	0.07004
L4	2.26333	3.90368	−0.62299
L5	4.27843	7.38996	−1.16689
G1	0.73679	1.36525	−0.10833

G2	1.32929	2.45730	−0.20127
G3	2.55920	4.73226	−0.38615
H1	0.10219	0.21348	0.00910
H2	0.57868	1.21853	0.06116
H3	1.07377	2.22680	0.07927
H4	2.05351	4.24177	0.13476
H5	2.07146	4.26211	0.11918
I1	0.05057	0.15588	0.05474
I2	0.25493	0.86849	0.35862
I3	0.59144	1.69505	0.51218
I4	1.18857	3.28373	0.90660

Table S4. Values of var_amp. var_max and var_min for each variable of FDMP model for SBR tests. Initial test data adapted from [46].

	**σ_1 (MPa)**			**σ_2 (Mpa)**			**σ_3 (Mpa)**		
**Test**	σ_{amp}	σ_{max}	σ_{min}	σ_{amp}	σ_{max}	σ_{min}	σ_{amp}	σ_{max}	σ_{min}
A 1	0.33747	0.68534	0.01039	0.08641	0.18113	0.00831	0.00020	0.00045	0.00005
A 2	0.42246	0.85687	0.01195	0.10170	0.21512	0.01172	0.00021	0.00048	0.00007
A 3	0.51128	1.03488	0.01232	0.11527	0.24609	0.01555	0.00021	0.00050	0.00009
A 4	0.69696	1.40151	0.00758	0.13654	0.29704	0.02397	0.00020	0.00051	0.00011
A 5	0.90694	1.80694	−0.00694	0.15121	0.33618	0.03376	0.00020	0.00048	0.00009
A 6	1.14586	2.25713	−0.03460	0.15941	0.36347	0.04465	0.00033	0.00052	−0.00013
A 7	1.40328	2.74493	−0.06163	0.16486	0.38410	0.05439	0.00064	0.00066	−0.00061
A 8	1.65842	3.23673	−0.08011	0.17026	0.40256	0.06204	0.00095	0.00077	−0.00113
B 9	0.27877	0.55119	−0.00634	0.00224	0.00041	−0.00408	0.18528	−0.01645	−0.38702
B 10	0.32147	0.63352	−0.00942	0.00278	0.00051	−0.00505	0.20313	−0.01970	−0.42595
B 11	0.36644	0.71987	−0.01301	0.00342	0.00073	−0.00612	0.21991	−0.02281	−0.46263
B 12	0.41407	0.81021	−0.01793	0.00391	0.00071	−0.00712	0.23538	−0.02686	−0.49762
B 13	0.46409	0.90478	−0.02340	0.00457	0.00087	−0.00826	0.25028	−0.03049	−0.53105
B 14	0.51685	1.00383	−0.02987	0.00549	0.00119	−0.00979	0.26454	−0.03423	−0.56331
B 15	0.57325	1.10872	−0.03779	0.00603	0.00121	−0.01084	0.27756	−0.03833	−0.59344
B 16	0.63260	1.21853	−0.04668	0.00694	0.00144	−0.01244	0.29029	−0.04224	−0.62281
B 17	0.69563	1.33433	−0.05692	0.00795	0.00167	−0.01422	0.30237	−0.04618	−0.65093
B 18	0.76171	1.45557	−0.06785	0.00970	0.00261	−0.01680	0.31439	−0.04971	−0.67849
B 19	0.82957	1.58052	−0.07861	0.01266	0.00426	−0.02105	0.32699	−0.05244	−0.70643
B 20	0.89878	1.70859	−0.08896	0.01640	0.00629	−0.02651	0.34015	−0.05453	−0.73483
B 21	0.96903	1.83897	−0.09909	0.02065	0.00834	−0.03297	0.35384	−0.05636	−0.76405
C 22	0.48808	0.98688	0.01071	0.02842	0.05156	−0.00528	0.00956	−0.00069	−0.01981
C 23	0.61751	1.25178	0.01675	0.10207	0.19483	−0.00930	0.00249	−0.00097	−0.00595
C 24	0.77093	1.55718	0.01531	0.15570	0.30585	−0.00555	0.00172	−0.00047	−0.00391
C 25	0.95736	1.91778	0.00307	0.19396	0.38931	0.00140	0.00184	0.00017	−0.00351
C 26	0.64488	1.28819	−0.00156	0.00531	0.00546	−0.00516	0.03405	−0.01036	−0.07846
C 27	0.74968	1.50815	0.00880	0.08889	0.15097	−0.02681	0.00832	−0.00130	−0.01794
C 28	0.87566	1.76415	0.01283	0.17028	0.30877	−0.03178	0.00322	−0.00218	−0.00863
C 29	1.03555	2.07710	0.00600	0.23516	0.43932	−0.03100	0.00341	−0.00083	−0.00764
C 30	0.85336	1.67638	−0.03034	0.00685	0.00668	−0.00702	0.06420	−0.03194	−0.16033
C 31	0.93897	1.86145	−0.01649	0.07615	0.11486	−0.03745	0.02275	−0.00077	−0.04628
C 32	1.04071	2.07457	−0.00685	0.17816	0.30276	−0.05355	0.00837	−0.00086	−0.01760
C 33	1.17099	2.33583	−0.00616	0.27190	0.48168	−0.06213	0.00395	−0.00153	−0.00942

D1 34	0.39185	0.82136	0.03766	0.00385	0.00044	−0.00726	0.21470	−0.07190	−0.50129
D1 35	0.41108	0.92143	0.09926	0.00437	0.00011	−0.00864	0.20316	−0.12543	−0.53174
D1 36	0.43178	1.02713	0.16356	0.00510	−0.00005	−0.01025	0.19201	−0.17615	−0.56018
D1 37	0.45418	1.13869	0.23033	0.00536	−0.00083	−0.01156	0.18145	−0.22449	−0.58739
D1 38	0.47804	1.25650	0.30042	0.00598	−0.00145	−0.01341	0.17186	−0.26976	−0.61348
D1 39	0.50411	1.38180	0.37357	0.00659	−0.00200	−0.01519	0.16254	−0.31259	−0.63767
D1 40	0.53126	1.51319	0.45066	0.00790	−0.00227	−0.01807	0.15464	−0.35269	−0.66197
D1 41	0.55824	1.64943	0.53294	0.01043	−0.00206	−0.02293	0.14863	−0.38967	−0.68694
D1 42	0.58475	1.79027	0.62078	0.01364	−0.00164	−0.02893	0.14395	−0.42418	−0.71208
D1 43	0.61003	1.93434	0.71427	0.01725	−0.00160	−0.03611	0.14086	−0.45663	−0.73835
D2 44	0.62621	1.28323	0.03081	0.00732	0.00115	−0.01349	0.26628	−0.10472	−0.63729
D2 45	0.67281	1.46162	0.11600	0.00890	0.00104	−0.01677	0.24709	−0.17605	−0.67023
D2 46	0.72037	1.65003	0.20928	0.01264	0.00214	−0.02313	0.23150	−0.24064	−0.70364
D2 47	0.76731	1.84633	0.31172	0.01770	0.00340	−0.03201	0.21955	−0.29920	−0.73831
D2 48	0.81263	2.04910	0.42384	0.02342	0.00420	−0.04263	0.21107	−0.35248	−0.77463
D2 49	0.85553	2.25706	0.54601	0.02936	0.00416	−0.05456	0.20583	−0.40128	−0.81293
D2 50	0.89524	2.46925	0.67877	0.03529	0.00329	−0.06728	0.20357	−0.44617	−0.85330
D2 51	0.93101	2.68447	0.82245	0.04104	0.00147	−0.08060	0.20401	−0.48789	−0.89591
D2 52	0.96206	2.90147	0.97735	0.04651	−0.00140	−0.09443	0.20690	−0.52716	−0.94095
D3 53	1.02532	1.99497	−0.05567	0.02517	0.01001	−0.04033	0.35430	−0.09239	−0.80099
D3 54	1.06625	2.14056	0.00805	0.02975	0.01116	−0.04834	0.34256	−0.14094	−0.82606
D3 55	1.10644	2.28878	0.07589	0.03433	0.01194	−0.05672	0.33233	−0.18705	−0.85170
D3 56	1.14511	2.43896	0.14874	0.03882	0.01237	−0.06527	0.32435	−0.22989	−0.87860
D3 57	1.18265	2.59112	0.22583	0.04322	0.01227	−0.07417	0.31804	−0.27038	−0.90646
D3 58	1.21902	2.74527	0.30723	0.04744	0.01174	−0.08315	0.31304	−0.30895	−0.93504
D3 59	1.25365	2.90090	0.39359	0.05150	0.01086	−0.09213	0.30988	−0.34507	−0.96483
D3 60	1.28655	3.05769	0.48459	0.05545	0.00953	−0.10137	0.30837	−0.37920	−0.99594
D3 61	1.31780	3.21581	0.58021	0.05917	0.00779	−0.11054	0.30793	−0.41192	−1.02777
D4 62	0.85442	1.88704	0.17820	0.02001	0.00570	−0.03431	0.26189	−0.23242	−0.75621
D4 63	0.78049	1.90366	0.34268	0.01931	0.00369	−0.03493	0.21678	−0.31494	−0.74851
D4 64	0.69919	1.91940	0.52102	0.01843	0.00135	−0.03552	0.17687	−0.38904	−0.74278
D4 65	0.61003	1.93434	0.71427	0.01725	−0.00160	−0.03611	0.14086	−0.45663	−0.73835
D4 66	0.51089	1.94748	0.92570	0.01610	−0.00482	−0.03703	0.10910	−0.51751	−0.73572
D4 67	0.40024	1.95812	1.15764	0.01464	−0.00888	−0.03816	0.08044	−0.57349	−0.73437
D5 68	0.64559	1.35795	0.06676	0.00776	0.00085	−0.01467	0.25802	−0.13572	−0.65176
D5 69	0.57804	1.37025	0.21417	0.00729	−0.00038	−0.01496	0.20765	−0.22866	−0.64397
D5 70	0.50411	1.38180	0.37357	0.00659	−0.00200	−0.01519	0.16254	−0.31259	−0.63767
D5 71	0.42281	1.39187	0.54624	0.00572	−0.00406	−0.01550	0.12263	−0.38821	−0.63347
D5 72	0.33365	1.40105	0.73374	0.00454	−0.00654	−0.01561	0.08662	−0.45689	−0.63013
D6 73	0.52173	1.12875	0.08528	0.00584	0.00039	−0.01128	0.23182	−0.13052	−0.59415
D6 74	0.45418	1.13869	0.23033	0.00536	−0.00083	−0.01156	0.18145	−0.22449	−0.58739
D6 75	0.38025	1.14770	0.38719	0.00467	−0.00244	−0.01178	0.13634	−0.30944	−0.58212
D6 76	0.29895	1.15524	0.55734	0.00379	−0.00436	−0.01195	0.09643	−0.38579	−0.57864
E1 77	0.34584	0.75451	0.06283	0.08543	0.19541	0.02456	0.00018	0.00046	0.00010
E1 78	0.34584	0.81626	0.12458	0.08148	0.20643	0.04347	0.00015	0.00047	0.00016
E1 79	0.34589	0.87798	0.18621	0.07763	0.21685	0.06159	0.00013	0.00047	0.00021
E1 80	0.34700	0.94087	0.24687	0.07444	0.22719	0.07830	0.00011	0.00047	0.00025
E1 81	0.34843	1.00439	0.30753	0.07100	0.23677	0.09476	0.00009	0.00047	0.00030
E1 82	0.34989	1.06809	0.36831	0.06773	0.24593	0.11047	0.00006	0.00046	0.00033
E1 83	0.35246	1.13332	0.42840	0.06485	0.25482	0.12513	0.00005	0.00046	0.00035

E1 84	0.35538	1.19945	0.48868	0.06178	0.26307	0.13951	0.00004	0.00045	0.00037
E1 85	0.35827	1.26591	0.54938	0.05894	0.27104	0.15317	0.00003	0.00045	0.00039
E1 86	0.36244	1.33443	0.60955	0.05625	0.27858	0.16608	0.00002	0.00045	0.00040
E2 87	0.52726	1.15925	0.10473	0.11030	0.26466	0.04407	0.00017	0.00050	0.00017
E2 88	0.53146	1.27384	0.21093	0.10168	0.27830	0.07493	0.00012	0.00049	0.00025
E2 89	0.53928	1.39359	0.31504	0.09341	0.29043	0.10360	0.00008	0.00047	0.00031
E2 90	0.54856	1.51628	0.41915	0.08599	0.30171	0.12972	0.00005	0.00045	0.00034
E2 91	0.56063	1.64429	0.52303	0.07844	0.31135	0.15447	0.00007	0.00046	0.00033
E2 92	0.57535	1.77769	0.62699	0.07176	0.32024	0.17673	0.00010	0.00048	0.00028
E2 93	0.59143	1.91538	0.73253	0.06516	0.32795	0.19763	0.00014	0.00049	0.00022
E2 94	0.61199	2.06179	0.83781	0.05849	0.33413	0.21714	0.00020	0.00051	0.00012
E2 95	0.63341	2.21296	0.94615	0.05233	0.33949	0.23482	0.00027	0.00053	0.00000
E3 96	0.95783	2.04403	0.12836	0.13701	0.35083	0.07680	0.00020	0.00048	0.00009
E3 97	0.99678	2.27802	0.28446	0.11796	0.35720	0.12128	0.00032	0.00057	−0.00007
E3 98	1.04024	2.52401	0.44352	0.10095	0.36204	0.16014	0.00047	0.00064	−0.00030
E3 99	1.08338	2.77599	0.60922	0.08629	0.36634	0.19376	0.00063	0.00068	−0.00057
E3 100	1.12534	3.03298	0.78231	0.07373	0.37026	0.22280	0.00078	0.00070	−0.00086
E3 101	1.16521	3.29443	0.96400	0.06306	0.37390	0.24778	0.00093	0.00069	−0.00118
E3 102	1.20204	3.55872	1.15463	0.05415	0.37750	0.26920	0.00108	0.00065	−0.00151
E3 103	1.23478	3.82506	1.35549	0.04691	0.38116	0.28734	0.00121	0.00058	−0.00185
E3 104	1.26236	4.09223	1.56752	0.04128	0.38505	0.30248	0.00134	0.00047	−0.00220
E3 105	1.28370	4.35842	1.79101	0.03724	0.38940	0.31492	0.00144	0.00032	−0.00256
E3 106	1.29786	4.62225	2.02653	0.03473	0.39436	0.32490	0.00152	0.00012	−0.00291
E4 107	1.07809	2.28299	0.12681	0.13882	0.36281	0.08518	0.00033	0.00055	−0.00010
E4 108	0.98610	2.28749	0.31528	0.11410	0.35656	0.12836	0.00033	0.00058	−0.00008
E4 109	0.89457	2.29331	0.50416	0.09255	0.35144	0.16634	0.00033	0.00058	−0.00007
E4 110	0.80239	2.30005	0.69526	0.07372	0.34726	0.19982	0.00033	0.00058	−0.00008
E4 111	0.70844	2.30812	0.89123	0.05729	0.34373	0.22915	0.00033	0.00056	−0.00009
E4 112	0.61150	2.31595	1.09295	0.04304	0.34097	0.25488	0.00033	0.00054	−0.00011
E4 113	0.51026	2.32386	1.30334	0.03083	0.33870	0.27704	0.00031	0.00049	−0.00014
E5 114	0.84265	1.82698	0.14169	0.13160	0.33597	0.07278	0.00014	0.00046	0.00018
E5 115	0.75821	1.82893	0.31251	0.10858	0.33105	0.11390	0.00012	0.00047	0.00023
E5 116	0.59003	1.83607	0.65600	0.07033	0.32386	0.18321	0.00012	0.00049	0.00025
E5 117	0.51240	1.83988	0.81508	0.05586	0.32152	0.20980	0.00012	0.00049	0.00025
E5 118	0.43314	1.84392	0.97764	0.04293	0.31960	0.23374	0.00012	0.00048	0.00024
E6 119	0.63615	1.41691	0.14460	0.11798	0.29725	0.06128	0.00015	0.00050	0.00021
E6 120	0.55926	1.41755	0.29903	0.09673	0.29358	0.10012	0.00009	0.00047	0.00030
E6 121	0.48287	1.41877	0.45303	0.07772	0.29064	0.13520	0.00005	0.00045	0.00036
E6 122	0.40642	1.42054	0.60770	0.06069	0.28827	0.16689	0.00003	0.00044	0.00039
E6 123	0.32931	1.42246	0.76384	0.04544	0.28650	0.19562	0.00003	0.00045	0.00040
E7 124	0.53896	1.18216	0.10424	0.11176	0.26798	0.04446	0.00017	0.00051	0.00017
E7 125	0.48467	1.18185	0.21252	0.09582	0.26538	0.07375	0.00012	0.00049	0.00025
E7 126	0.43100	1.18211	0.32012	0.08130	0.26334	0.10074	0.00008	0.00047	0.00031
E7 127	0.37752	1.18258	0.42753	0.06799	0.26167	0.12569	0.00005	0.00046	0.00035
E7 128	0.32416	1.18333	0.53502	0.05530	0.26002	0.14943	0.00003	0.00045	0.00039

	ε_1			ε_2			ε_3		
**Test**	ε_{amp}	ε_{max}	ε_{min}	ε_{amp}	ε_{max}	ε_{min}	ε_{amp}	ε_{max}	ε_{min}
A 1	0.15777	0.32118	0.00564	0.04024	0.00026	−0.08023	0.11753	−0.00589	−0.24095
A 2	0.19564	0.39864	0.00737	0.05175	0.00066	−0.10283	0.14389	−0.00803	−0.29581

A 3	0.23361	0.47650	0.00928	0.06394	0.00116	−0.12671	0.16967	−0.01044	−0.34978
A 4	0.30607	0.62592	0.01378	0.08898	0.00221	−0.17575	0.21709	−0.01598	−0.45017
A 5	0.37498	0.76994	0.01999	0.11505	0.00310	−0.22699	0.25993	−0.02309	−0.54295
A 6	0.43711	0.90299	0.02876	0.14071	0.00338	−0.27805	0.29640	−0.03214	−0.62494
A 7	0.49640	1.03030	0.03750	0.16624	0.00324	−0.32924	0.33017	−0.04074	−0.70107
A 8	0.55517	1.15481	0.04447	0.19153	0.00306	−0.38001	0.36364	−0.04753	−0.77480
B 9	0.19872	0.40188	0.00444	0.01554	0.00348	−0.02760	0.18318	−0.00792	−0.37428
B 10	0.22388	0.45280	0.00505	0.01927	0.00427	−0.03427	0.20460	−0.00932	−0.41852
B 11	0.24898	0.50353	0.00557	0.02334	0.00510	−0.04159	0.22564	−0.01066	−0.46194
B 12	0.27358	0.55383	0.00667	0.02754	0.00588	−0.04920	0.24604	−0.01255	−0.50464
B 13	0.29813	0.60385	0.00758	0.03193	0.00667	−0.05719	0.26620	−0.01425	−0.54666
B 14	0.32261	0.65377	0.00855	0.03656	0.00752	−0.06561	0.28604	−0.01607	−0.58816
B 15	0.34595	0.70212	0.01023	0.04094	0.00807	−0.07382	0.30500	−0.01829	−0.62830
B 16	0.36923	0.75036	0.01191	0.04549	0.00862	−0.08237	0.32373	−0.02052	−0.66799
B 17	0.39183	0.79763	0.01397	0.04996	0.00900	−0.09091	0.34187	−0.02298	−0.70672
B 18	0.41467	0.84478	0.01544	0.05477	0.00974	−0.09979	0.35990	−0.02518	−0.74498
B 19	0.43874	0.89341	0.01593	0.06032	0.01090	−0.10974	0.37842	−0.02683	−0.78367
B 20	0.46382	0.94330	0.01565	0.06642	0.01234	−0.12050	0.39740	−0.02799	−0.82280
B 21	0.48973	0.99456	0.01510	0.07291	0.01385	−0.13196	0.41682	−0.02895	−0.86259
C 22	0.25426	0.52237	0.01385	0.12137	−0.00120	−0.24394	0.13289	−0.01265	−0.27843
C 23	0.29765	0.61444	0.01913	0.11278	−0.00968	−0.23523	0.18488	−0.00945	−0.37920
C 24	0.34764	0.71984	0.02456	0.11527	−0.01089	−0.24142	0.23237	−0.01367	−0.47842
C 25	0.40088	0.83284	0.03109	0.12564	−0.01084	−0.26213	0.27523	−0.02025	−0.57072
C 26	0.33225	0.68463	0.02012	0.14776	0.00139	−0.29414	0.18449	−0.02151	−0.39049
C 27	0.35989	0.74691	0.02713	0.15874	−0.01565	−0.33313	0.20115	−0.01148	−0.41379
C 28	0.39422	0.82256	0.03411	0.14590	−0.02175	−0.31355	0.24832	−0.01236	−0.50901
C 29	0.43366	0.90902	0.04170	0.14249	−0.02349	−0.30847	0.29117	−0.01822	−0.60055
C 30	0.40845	0.84675	0.02985	0.15972	−0.00029	−0.31973	0.24873	−0.02956	−0.52702
C 31	0.42441	0.88702	0.03820	0.19296	−0.01387	−0.39980	0.23145	−0.02432	−0.48722
C 32	0.44624	0.93898	0.04649	0.18111	−0.03117	−0.39339	0.26513	−0.01533	−0.54559
C 33	0.47320	1.00148	0.05507	0.16740	−0.03540	−0.37021	0.30580	−0.01967	−0.63127
D1 34	0.25535	0.56019	0.04948	0.02725	0.00412	−0.05038	0.22810	−0.05360	−0.50981
D1 35	0.25544	0.61120	0.10033	0.03086	0.00203	−0.05969	0.22458	−0.10236	−0.55151
D1 36	0.25521	0.66169	0.15127	0.03430	−0.00086	−0.06947	0.22091	−0.15041	−0.59222
D1 37	0.25367	0.71081	0.20348	0.03687	−0.00519	−0.07892	0.21680	−0.19829	−0.63190
D1 38	0.25199	0.75954	0.25556	0.03918	−0.01032	−0.08869	0.21281	−0.24523	−0.67085
D1 39	0.24945	0.80665	0.30774	0.04104	−0.01622	−0.09830	0.20841	−0.29153	−0.70835
D1 40	0.24711	0.85380	0.35958	0.04276	−0.02270	−0.10821	0.20435	−0.33688	−0.74559
D1 41	0.24613	0.90251	0.41025	0.04488	−0.02945	−0.11922	0.20125	−0.38081	−0.78330
D1 42	0.24605	0.95201	0.45991	0.04725	−0.03641	−0.13090	0.19881	−0.42350	−0.82111
D1 43	0.24685	1.00286	0.50916	0.04966	−0.04391	−0.14324	0.19719	−0.46525	−0.85963
D2 44	0.35292	0.77835	0.07252	0.04694	0.00575	−0.08813	0.30598	−0.07826	−0.69022
D2 45	0.34998	0.84503	0.14507	0.05181	0.00126	−0.10236	0.29817	−0.14633	−0.74267
D2 46	0.34884	0.91365	0.21597	0.05710	−0.00409	−0.11828	0.29174	−0.21188	−0.79537
D2 47	0.34936	0.98455	0.28582	0.06249	−0.01068	−0.13567	0.28687	−0.27514	−0.84888
D2 48	0.35120	1.05748	0.35508	0.06769	−0.01870	−0.15408	0.28351	−0.33637	−0.90340
D2 49	0.35414	1.13234	0.42406	0.07252	−0.02819	−0.17323	0.28162	−0.39587	−0.95911
D2 50	0.35809	1.20882	0.49263	0.07693	−0.03893	−0.19280	0.28116	−0.45370	−1.01603
D2 51	0.36307	1.28699	0.56085	0.08096	−0.05076	−0.21269	0.28211	−0.51009	−1.07430
D2 52	0.36916	1.36704	0.62871	0.08470	−0.06350	−0.23290	0.28446	−0.56522	−1.13414

D3 53	0.50419	1.05833	0.04995	0.07947	0.01303	−0.14590	0.42472	−0.06299	−0.91243
D3 54	0.50687	1.11226	0.09852	0.08562	0.01098	−0.16025	0.42125	−0.10950	−0.95200
D3 55	0.50978	1.16682	0.14727	0.09146	0.00804	−0.17489	0.41831	−0.15530	−0.99193
D3 56	0.51290	1.22184	0.19604	0.09674	0.00403	−0.18945	0.41616	−0.20007	−1.03239
D3 57	0.51627	1.27771	0.24516	0.10162	−0.00100	−0.20425	0.41465	−0.24416	−1.07346
D3 58	0.51974	1.33399	0.29451	0.10613	−0.00682	−0.21908	0.41362	−0.28768	−1.11491
D3 59	0.52341	1.39066	0.34383	0.11012	−0.01349	−0.23373	0.41329	−0.33034	−1.15693
D3 60	0.52741	1.44815	0.39332	0.11374	−0.02098	−0.24846	0.41367	−0.37234	−1.19969
D3 61	0.53149	1.50593	0.44295	0.11701	−0.02909	−0.26311	0.41448	−0.41387	−1.24283
D4 62	0.40112	1.00583	0.20360	0.06889	−0.00085	−0.13863	0.33223	−0.20274	−0.86720
D4 63	0.34979	1.00525	0.30566	0.06403	−0.01282	−0.14088	0.28577	−0.29283	−0.86437
D4 64	0.29834	1.00424	0.40756	0.05749	−0.02736	−0.14234	0.24085	−0.38020	−0.86190
D4 65	0.24685	1.00286	0.50916	0.04966	−0.04391	−0.14324	0.19719	−0.46525	−0.85963
D4 66	0.19648	1.00182	0.60886	0.04105	−0.06173	−0.14383	0.15543	−0.54714	−0.85799
D4 67	0.14745	1.00123	0.70634	0.03202	−0.08026	−0.14431	0.11542	−0.62608	−0.85692
D5 68	0.35157	0.80701	0.10386	0.04893	0.00376	−0.09409	0.30265	−0.10762	−0.71292
D5 69	0.30077	0.80705	0.20550	0.04590	−0.00471	−0.09651	0.25487	−0.20079	−0.71054
D5 70	0.24945	0.80665	0.30774	0.04104	−0.01622	−0.09830	0.20841	−0.29153	−0.70835
D5 71	0.19800	0.80617	0.41018	0.03450	−0.03045	−0.09945	0.16350	−0.37973	−0.70672
D5 72	0.14651	0.80517	0.51215	0.02667	−0.04670	−0.10004	0.11984	−0.46546	−0.70513
D6 73	0.30447	0.71056	0.10162	0.03989	0.00306	−0.07672	0.26458	−0.10469	−0.63384
D6 74	0.25367	0.71081	0.20348	0.03687	−0.00519	−0.07892	0.21680	−0.19829	−0.63190
D6 75	0.20235	0.71069	0.30600	0.03200	−0.01651	−0.08052	0.17034	−0.28949	−0.63017
D6 76	0.15089	0.71037	0.40859	0.02547	−0.03055	−0.08148	0.12543	−0.37804	−0.62889
E1 77	0.16103	0.35274	0.03067	0.04199	−0.00529	−0.08928	0.11904	−0.02538	−0.26346
E1 78	0.16015	0.38043	0.06012	0.04292	−0.01189	−0.09773	0.11724	−0.04823	−0.28270
E1 79	0.15930	0.40799	0.08940	0.04382	−0.01867	−0.10632	0.11548	−0.07073	−0.30168
E1 80	0.15855	0.43549	0.11839	0.04459	−0.02576	−0.11495	0.11396	−0.09263	−0.32054
E1 81	0.15772	0.46278	0.14734	0.04542	−0.03292	−0.12375	0.11231	−0.11442	−0.33903
E1 82	0.15694	0.48998	0.17611	0.04621	−0.04025	−0.13266	0.11073	−0.13586	−0.35732
E1 83	0.15615	0.51700	0.20470	0.04689	−0.04783	−0.14162	0.10926	−0.15687	−0.37538
E1 84	0.15530	0.54382	0.23323	0.04762	−0.05549	−0.15073	0.10768	−0.17774	−0.39309
E1 85	0.15449	0.57055	0.26157	0.04829	−0.06332	−0.15990	0.10620	−0.19825	−0.41065
E1 86	0.15357	0.59698	0.28984	0.04888	−0.07136	−0.16912	0.10469	−0.21847	−0.42786
E2 87	0.23751	0.52920	0.05419	0.06737	−0.00897	−0.14372	0.17013	−0.04522	−0.38548
E2 88	0.23531	0.57598	0.10536	0.06938	−0.02105	−0.15981	0.16593	−0.08430	−0.41617
E2 89	0.23288	0.62198	0.15622	0.07125	−0.03368	−0.17618	0.16163	−0.12254	−0.44580
E2 90	0.23048	0.66750	0.20654	0.07288	−0.04692	−0.19268	0.15760	−0.15963	−0.47482
E2 91	0.22771	0.71207	0.25665	0.07445	−0.06052	−0.20941	0.15326	−0.19613	−0.50266
E2 92	0.22472	0.75577	0.30634	0.07565	−0.07480	−0.22610	0.14907	−0.23154	−0.52968
E2 93	0.22151	0.79865	0.35562	0.07675	−0.08939	−0.24288	0.14476	−0.26624	−0.55577
E2 94	0.21750	0.83986	0.40487	0.07756	−0.10445	−0.25957	0.13994	−0.30042	−0.58029
E2 95	0.21333	0.88014	0.45348	0.07813	−0.11992	−0.27618	0.13520	−0.33356	−0.60396
E3 96	0.37699	0.84502	0.09104	0.12117	−0.01352	−0.25586	0.25582	−0.07752	−0.58916
E3 97	0.36785	0.90810	0.17240	0.12417	−0.03413	−0.28246	0.24368	−0.13827	−0.62564
E3 98	0.35846	0.96909	0.25217	0.12643	−0.05596	−0.30883	0.23202	−0.19621	−0.66026
E3 99	0.34958	1.02914	0.32998	0.12812	−0.07881	−0.33505	0.22146	−0.25117	−0.69409
E3 100	0.34120	1.08836	0.40597	0.12929	−0.10253	−0.36111	0.21191	−0.30344	−0.72725
E3 101	0.33335	1.14681	0.48011	0.13001	−0.12695	−0.38697	0.20334	−0.35315	−0.75984
E3 102	0.32612	1.20472	0.55248	0.13034	−0.15195	−0.41264	0.19578	−0.40053	−0.79209

E3 103	0.31965	1.26220	0.62291	0.13037	−0.17737	−0.43810	0.18928	−0.44554	−0.82410
E3 104	0.31412	1.31945	0.69122	0.13018	−0.20304	−0.46339	0.18394	−0.48818	−0.85607
E3 105	0.30977	1.37685	0.75731	0.12988	−0.22880	−0.48856	0.17989	−0.52851	−0.88830
E3 106	0.30684	1.43468	0.82100	0.12959	−0.25449	−0.51367	0.17725	−0.56651	−0.92101
E4 107	0.40510	0.91189	0.10168	0.13353	−0.01515	−0.28221	0.27157	−0.08654	−0.62968
E4 108	0.36174	0.91010	0.18661	0.12281	−0.03799	−0.28361	0.23893	−0.14862	−0.62649
E4 109	0.31906	0.90847	0.27036	0.11134	−0.06206	−0.28474	0.20772	−0.20831	−0.62374
E4 110	0.27698	0.90696	0.35301	0.09920	−0.08721	−0.28562	0.17777	−0.26580	−0.62134
E4 111	0.23550	0.90536	0.43436	0.08648	−0.11330	−0.28625	0.14902	−0.32107	−0.61911
E4 112	0.19469	0.90389	0.51450	0.07324	−0.14018	−0.28666	0.12145	−0.37431	−0.61722
E4 113	0.15470	0.90240	0.59300	0.05959	−0.16771	−0.28690	0.09511	−0.42528	−0.61550
E5 114	0.34461	0.77761	0.08838	0.10826	−0.01391	−0.23043	0.23635	−0.07447	−0.54718
E5 115	0.30478	0.77638	0.16681	0.09851	−0.03456	−0.23158	0.20627	−0.13226	−0.54481
E5 116	0.22681	0.77428	0.32066	0.07717	−0.07888	−0.23323	0.14963	−0.24178	−0.54105
E5 117	0.19204	0.77345	0.38937	0.06676	−0.10022	−0.23373	0.12529	−0.28915	−0.53972
E5 118	0.15770	0.77266	0.45726	0.05597	−0.12217	−0.23411	0.10173	−0.33508	−0.53855
E6 119	0.27735	0.63219	0.07748	0.08256	−0.01330	−0.17842	0.19479	−0.06418	−0.45377
E6 120	0.24108	0.63134	0.14919	0.07377	−0.03175	−0.17928	0.16731	−0.11744	−0.45206
E6 121	0.20528	0.63063	0.22007	0.06446	−0.05107	−0.17998	0.14082	−0.16900	−0.45064
E6 122	0.16991	0.63001	0.29019	0.05467	−0.07121	−0.18055	0.11524	−0.21898	−0.44946
E6 123	0.13495	0.62951	0.35960	0.04445	−0.09207	−0.18098	0.09050	−0.26753	−0.44853
E7 124	0.24219	0.53869	0.05431	0.06896	−0.00888	−0.14680	0.17323	−0.04542	−0.39189
E7 125	0.21657	0.53812	0.10499	0.06304	−0.02132	−0.14740	0.15353	−0.08366	−0.39072
E7 126	0.19125	0.53766	0.15516	0.05681	−0.03428	−0.14789	0.13444	−0.12088	−0.38977
E7 127	0.16619	0.53727	0.20489	0.05029	−0.04771	−0.14829	0.11590	−0.15718	−0.38898
E7 128	0.14127	0.53688	0.25435	0.04363	−0.06143	−0.14870	0.09763	−0.19292	−0.38819

	**SED (MPa)**		
**Test**	W_{amp}	W_{max}	W_{min}
A 1	0.05155	0.09162	−0.01147
A 2	0.07951	0.14082	−0.01821
A 3	0.11396	0.20136	−0.02656
A 4	0.19907	0.35106	−0.04708
A 5	0.30688	0.54133	−0.07243
A 6	0.43212	0.76378	−0.10045
A 7	0.56581	1.00725	−0.12438
A 8	0.69833	1.25435	−0.14231
B 9	0.09223	0.16227	−0.02219
B 10	0.11614	0.20423	−0.02805
B 11	0.14285	0.25171	−0.03400
B 12	0.17287	0.30383	−0.04190
B 13	0.20495	0.36042	−0.04948
B 14	0.23925	0.42091	−0.05759
B 15	0.27683	0.48699	−0.06666
B 16	0.31594	0.55619	−0.07568
B 17	0.35717	0.62935	−0.08499
B 18	0.39948	0.70517	−0.09380
B 19	0.44180	0.78209	−0.10151
B 20	0.48411	0.85992	−0.10830

B 21	0.52642	0.93800	−0.11485
C 22	0.14377	0.25388	−0.03366
C 23	0.20619	0.36362	−0.04875
C 24	0.29130	0.51332	−0.06927
C 25	0.39911	0.70359	−0.09462
C 26	0.25649	0.45204	−0.06095
C 27	0.31891	0.56177	−0.07604
C 28	0.40401	0.71148	−0.09655
C 29	0.51182	0.90175	−0.12190
C 30	0.40872	0.72097	−0.09647
C 31	0.47082	0.83015	−0.11149
C 32	0.55624	0.98041	−0.13207
C 33	0.66405	1.17068	−0.15742
D1 34	0.17134	0.31117	−0.03151
D1 35	0.19907	0.37589	−0.02226
D1 36	0.22673	0.44630	−0.00717
D1 37	0.25391	0.52106	0.01324
D1 38	0.27993	0.60009	0.04023
D1 39	0.30581	0.68457	0.07296
D1 40	0.32947	0.77101	0.11207
D1 41	0.35008	0.85846	0.15829
D1 42	0.36848	0.94770	0.21074
D1 43	0.38408	1.03695	0.26878
D2 44	0.32999	0.60021	−0.05976
D2 45	0.38037	0.72078	−0.03996
D2 46	0.42547	0.84486	−0.00608
D2 47	0.46498	0.97100	0.04104
D2 48	0.49893	1.09834	0.10049
D2 49	0.52738	1.22607	0.17131
D2 50	0.55042	1.35371	0.25287
D2 51	0.56818	1.48057	0.34420
D2 52	0.58088	1.60596	0.44420
D3 53	0.56772	1.03135	−0.10409
D3 54	0.60569	1.12497	−0.08640
D3 55	0.64136	1.21952	−0.06319
D3 56	0.67328	1.31374	−0.03281
D3 57	0.70250	1.40815	0.00315
D3 58	0.72945	1.50301	0.04411
D3 59	0.75311	1.59737	0.09115
D3 60	0.77373	1.69097	0.14352
D3 61	0.79213	1.78464	0.20039
D4 62	0.50382	0.98733	−0.02030
D4 63	0.47526	1.00729	0.05676
D4 64	0.43481	1.02355	0.15392
D4 65	0.38408	1.03695	0.26878
D4 66	0.32189	1.04633	0.40255
D4 67	0.24991	1.05227	0.55246
D5 68	0.35150	0.65073	−0.05227
D5 69	0.33436	0.66902	0.00030
D5 70	0.30581	0.68457	0.07296

D5 71	0.26535	0.69613	0.16542
D5 72	0.21463	0.70556	0.27631
D6 73	0.27105	0.50547	−0.03664
D6 74	0.25391	0.52106	0.01324
D6 75	0.22536	0.53387	0.08316
D6 76	0.18491	0.54314	0.17333
E1 77	0.06005	0.11012	−0.00999
E1 78	0.06916	0.13064	−0.00769
E1 79	0.07808	0.15250	−0.00367
E1 80	0.08622	0.17534	0.00290
E1 81	0.09512	0.20031	0.01008
E1 82	0.10365	0.22636	0.01907
E1 83	0.11183	0.25382	0.03016
E1 84	0.12060	0.28323	0.04204
E1 85	0.12883	0.31348	0.05582
E1 86	0.13707	0.34547	0.07134
E2 87	0.13667	0.25013	−0.02321
E2 88	0.15881	0.30258	−0.01505
E2 89	0.18176	0.36079	−0.00273
E2 90	0.20321	0.42204	0.01561
E2 91	0.22575	0.48880	0.03731
E2 92	0.24647	0.55851	0.06556
E2 93	0.26709	0.63195	0.09776
E2 94	0.28790	0.71004	0.13424
E2 95	0.30702	0.79051	0.17646
E3 96	0.36390	0.66321	−0.06458
E3 97	0.41775	0.79257	−0.04292
E3 98	0.46738	0.92739	−0.00737
E3 99	0.51124	1.06465	0.04216
E3 100	0.54941	1.20357	0.10474
E3 101	0.58195	1.34346	0.17957
E3 102	0.60890	1.48338	0.26559
E3 103	0.63037	1.62279	0.36206
E3 104	0.64654	1.76111	0.46802
E3 105	0.65773	1.89772	0.58227
E3 106	0.66436	2.03236	0.70364
E4 107	0.42921	0.78325	−0.07518
E4 108	0.41793	0.79961	−0.03625
E4 109	0.39961	0.81410	0.01488
E4 110	0.37443	0.82674	0.07787
E4 111	0.34249	0.83785	0.15287
E4 112	0.30384	0.84675	0.23906
E4 113	0.25859	0.85404	0.33687
E5 114	0.30431	0.55629	−0.05233
E5 115	0.29471	0.56868	−0.02074
E5 116	0.25789	0.58898	0.07321
E5 117	0.23349	0.59619	0.12921
E5 118	0.20454	0.60233	0.19324
E6 119	0.19664	0.36187	−0.03140
E6 120	0.18855	0.37069	−0.00640

E6 121	0.17565	0.37827	0.02698
E6 122	0.15806	0.38485	0.06872
E6 123	0.13586	0.39007	0.11834
E7 124	0.14181	0.25926	−0.02436
E7 125	0.13824	0.26502	−0.01147
E7 126	0.13188	0.26993	0.00618
E7 127	0.12285	0.27412	0.02842
E7 128	0.11248	0.27848	0.05352
